# Quiescence status of glioblastoma stem-like cells involves remodelling of Ca^2+^ signalling and mitochondrial shape

**DOI:** 10.1038/s41598-018-28157-8

**Published:** 2018-06-27

**Authors:** Francisco J. Aulestia, Isabelle Néant, Jihu Dong, Jacques Haiech, Marie-Claude Kilhoffer, Marc Moreau, Catherine Leclerc

**Affiliations:** 1 Centre de Biologie du Développement (CBD), Centre de Biologie Intégrative (CBI), Université de Toulouse, CNRS, UPS, F-31062 Toulouse, France; 2 Laboratoire d’Excellence Medalis, Université de Strasbourg, CNRS, LIT UMR 7200, F-67000 Strasbourg, France

## Abstract

Quiescence is a reversible cell-cycle arrest which allows cancer stem-like cells to evade killing following therapies. Here, we show that proliferating glioblastoma stem-like cells (GSLCs) can be induced and maintained in a quiescent state by lowering the extracellular pH. Through RNAseq analysis we identified Ca^2+^ signalling genes differentially expressed between proliferating and quiescent GSLCs. Using the bioluminescent Ca^2+^ reporter EGFP-aequorin we observed that the changes in Ca^2+^ homeostasis occurring during the switch from proliferation to quiescence are controlled through store-operated channels (SOC) since inhibition of SOC drives proliferating GSLCs to quiescence. We showed that this switch is characterized by an increased capacity of GSLCs’ mitochondria to capture Ca^2+^ and by a dramatic and reversible change of mitochondrial morphology from a tubular to a donut shape. Our data suggest that the remodelling of the Ca^2+^ homeostasis and the reshaping of mitochondria might favours quiescent GSLCs’ survival and their aggressiveness in glioblastoma.

## Introduction

Multiform glioblastoma (GBM) is the most aggressive brain tumours with very poor prognosis. Despite a combination of surgical resection, radiotherapy and temozolomide (TMZ)-based chemotherapy, more than 90% of the patients show recurrence and the mean survival period rarely exceeds 2 years^1^. According to the cancer stem cell model, the GBM lethality is due to a small sub-population of tumour cells with stem-like properties, called Glioblastoma Stem-Like Cells (GSLCs). The GSLCs have been further characterized as slow-cycling or relatively quiescent cells^2^, identified *in vivo* in a mouse model of glioblastoma^3^ and in human glioblastoma tumors^4^. These quiescent GSLCs are highly resistant to TMZ treatment^5^.

Quiescence is a cell-cycle arrest state which differs from the one observed in differentiation or senescence by the fact that it is reversible. Transcriptional profiling data reveals that quiescent stem cells are characterized by a common gene signature with the down-regulation of genes associated with cell-cycle progression (i.e. *CCNA2*, *CCNB1* and *CCNE2*) and the upregulation of genes classified as tumour suppressors, including the cyclin-dependent kinase inhibitor p21 (*CDKN1A*) and the G0/G1 switch gene 2 (*G0S2*)^6,7^. These data also show that quiescence is a G_0_ phase and not a prolonged G_1_ phase^8^. Furthermore, quiescence is actively regulated by signals provided by the stem cell microenvironment. In glioblastoma tumours, quiescent stem-like tumour cells are found close to necrotic tissues, in specific niches characterized by an hypoxic^4,5,9^ and acidic microenvironment^10,11^. The role of the microenvironment in the control of GSLCs quiescence is still poorly understood.

Considering that quiescence represents a strategy for GSLCs to evade killing, it is of utmost importance to better characterize the quiescent GSLCs and to understand what governs the transition from a proliferative to a quiescence state. Here, we performed transcriptomic analysis using RNAseq to establish the RNA signatures of proliferative and quiescent GSLCs. We showed that genes involved in Ca^2+^ signalling are modulated in GSLCs and we explored the causal role of Ca^2+^ during this transition. Our data points out the reversible remodelling of mitochondrial morphology from tubular to donut shape, associated with an increased capacity of mitochondria to capture Ca^2+^ and with the modification of the kinetics of Ca^2+^ influx through SOC. The remodelling of mitochondrial morphology was also observed in an *ex-vivo* tumour model consisting of large glioblastoma tumorospheres. Our data suggest that the remodelling of the Ca^2+^ homeostasis and the reshaping of mitochondria during the transition from proliferation to quiescence constitute a protective mechanism that favours survival and aggressiveness of GSLCs.

## Results

### *In vitro* induction of quiescence in GSLCs

**Figure 1:**
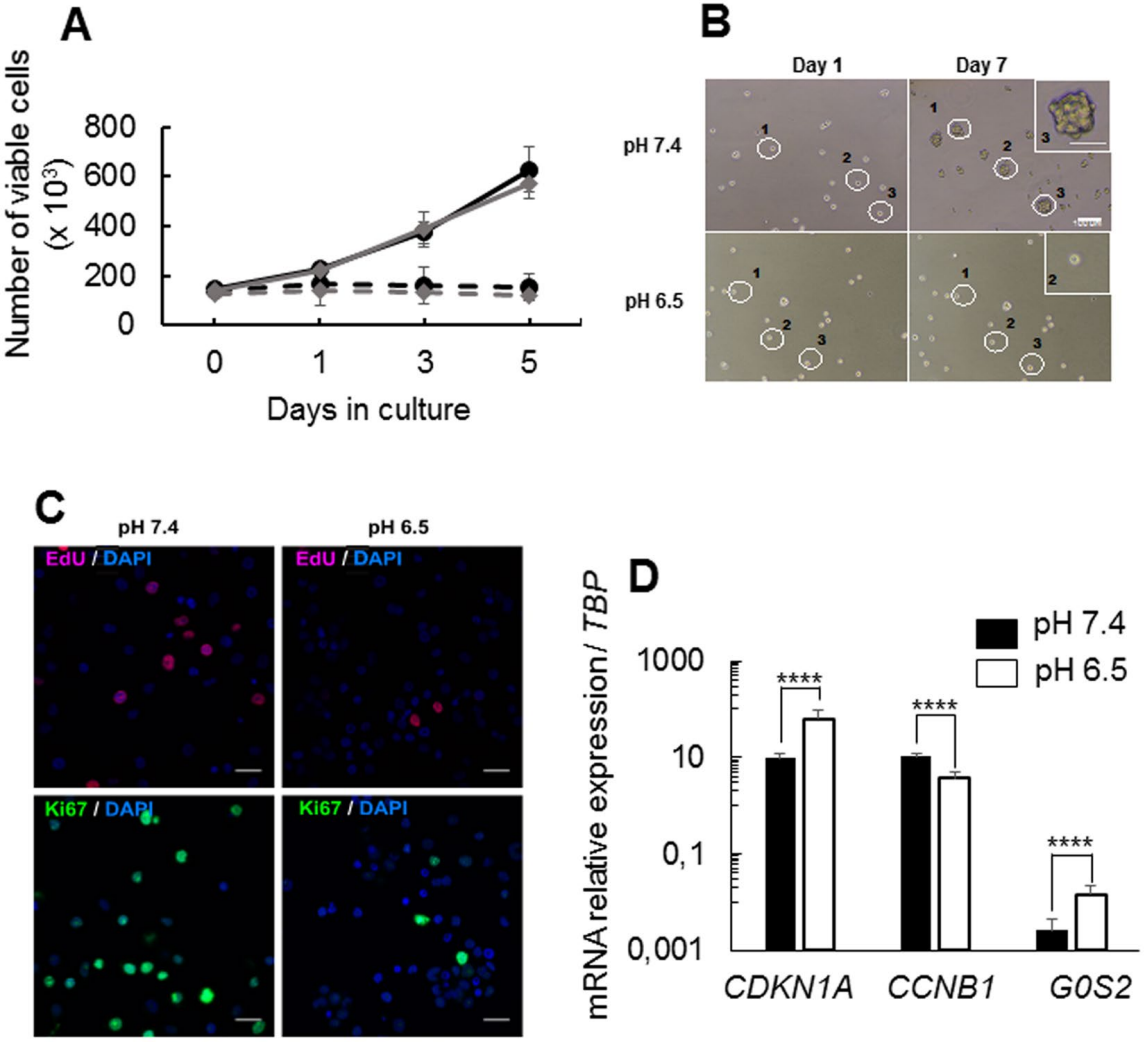
*In vitro* induction of quiescence in GSLCs. (**A**) Cell proliferation measured by counting the number of viable cells over 5 days in NS34 medium at pH 7.4 for TG1 and TG1_C1 cells (black and grey lines respectively) and in NS34 at pH 6.5 for TG1 and TG1_C1 cells (dotted black and gray lines respectively). Each measure was done in triplicates and with 3 independent experiments. (**B**) TG1 cells were plated in semi-solid agar medium at pH 7.5 or pH 6.5 to assess their ability to form secondary spheres. Shown are the same fields taken at Day 1 and Day 7 in the 2 culture conditions. Only single cells culture at pH 7.4 (white circles) are able to proliferate and to form a secondary spheres (Inset). (**C**) EdU incorporation (upper panels) and Ki67 positive cells (lower panels) at pH 7.4 and pH 6.5 for TG1 cells. (**D**) Expression of *CDKN1A*, *CCNB1* and *G0S2* in TG1 cells in NS34 at pH7.4 compared to TG1 cells in NS34 at pH 6.5, was assessed by QRT-PCR after 5 days of culture. Results are given relative to *TBP* (TATA-Box Binding Protein) expression level. Error bars are derived from 11 independent experiments. Pictures were taken with a 20X 0.40 N.A. objective on Nikon eclipse TS100 microscope. Scale bars: 100 µm in B and 50 µm in Inset, 10 µm in C.

TG1 and TG1_C1 cells are human GSLCs previously characterized^12,13^. Previous data showed that TG1 and TG1_C1 cells cultured without medium renewal during 9 days stopped proliferation. This cell-cycle arrest was shown to be reversible, to maintain cells’ stemness and differentiation properties and is not accompanied by cell senescence^13^. Interestingly, this culture condition induced an acidification of the medium from pH 7.4 to pH 6.6 which correlates with a decrease in EdU incorporation suggesting that the cells adopt a quiescent phenotype^14^. In order to further characterize this quiescent state, GSLCs were seeded in NS34 medium at pH 7.4 and 6.5 and cell proliferation and viability analysed during 5 days by cell counting and trypan blue exclusion respectively. In proliferating medium (NS34 medium, pH 7.4) the number of TG1 and TG1_C1 cells increased by about 4-fold while at pH 6.5, proliferation rapidly stopped and by day 5 the number of cells was not significantly different from day 0 (Fig. 1A). Analysis of cell viability indicates that lowering extracellular pH (pH_e_) to 6.5 does not induce cell death (Supplementary Fig. S1). The ability of TG1 cells to form new spheres was evaluated by seeding mechanically dissociated TG1 cells in semi-solid agar medium at pH 7.4 or pH 6.5. Isolated TG1 cells in pH 7.4 medium are able to form spheres of about 40 µm diameter (n = 39.5 µm + 8.8, n = 12), while at pH 6.5, isolated TG1 cells never formed spheres (Fig. 1B). To further confirm that acidic pH_e_ induces proliferation-arrest we measured the number of cells incorporating EdU. The percentage of cells in the S phase decreased drastically in cells kept at pH 6.5 compared to pH 7.4 (at pH 7.4, 39.1% ± 8.9%; at pH 6.5, 4.1% ± 0.8%, p < 0.001, 3 independent experiments), indicating that cells have stopped proliferating (Fig. 1C and Supplementary Fig. S1B). This is confirmed by immunostaining of Ki67 protein (Fig. 1C and Supplementary Fig. S1B), showing that at pH 6.5 TG1 cells had withdrawn from the cell cycle into the G_0_ phase. Interestingly, the modification of culture conditions from pH 7.4 to pH 6.5 did not alter the expression of the stemness markers, NANOG, OLIG2 and SOX2, known to promote and to maintain stemness of GSLCs^15^ (Supplementary Fig. S1C). To further demonstrate that the TG1 cells grown at pH 6.5 are in a quiescent state, we analysed the mRNA expression levels of (i) *CDKN1A*, a cyclin-dependent kinase inhibitor, expressed in quiescent cells and involved in maintenance of the quiescent state^16^, (ii) *CCNB1* (cyclin B1) down-regulated in quiescent cells^8^ and (iii) *G0S2* (G0/G1 Switch 2 gene), encoding a cytosolic protein which promotes quiescence of hematopoietic stem cells^7^. As expected, the transition from proliferative to quiescent GSLCs is associated with the decrease in the mRNA level for *CCNB1* and the increase in mRNA level for *CDKN1A* and *G0S2* (Fig. 1D). Another feature of quiescence is its reversibility^6^. Quiescent TG1 cells cultured during 5 days at pH 6.5 were transferred to freshly prepared NS34 medium at pH 7.4. Compared to their counterparts kept at pH 6.5, these cells rapidly resumed proliferation as shown by EdU incorporation profile, the number of Ki67 expressing cells, the expression of cell cycle markers and of *G0S2* (Supplementary Fig. S2). In order to extend our results to other cell lines, we tested the capacity of BTIC25 GSLCs, from an independent origin^17^, to be induced toward quiescence. Similarly to TG1 and TG1_C1 cells, BTIC25 cells cultured 5 days in NS34 medium at pH 6.5 stopped proliferating and showed an increased expression of *CDKN1A* and *G0S2* and a decrease in *CCNB1* (supplementary Fig. S3). Altogether these results strongly suggest that low pH_e_ activates a cellular program that induces quiescence of GSLCs while preserving stemness properties.

### RNA signatures of proliferative and quiescent GSLCs

**Figure 2:**
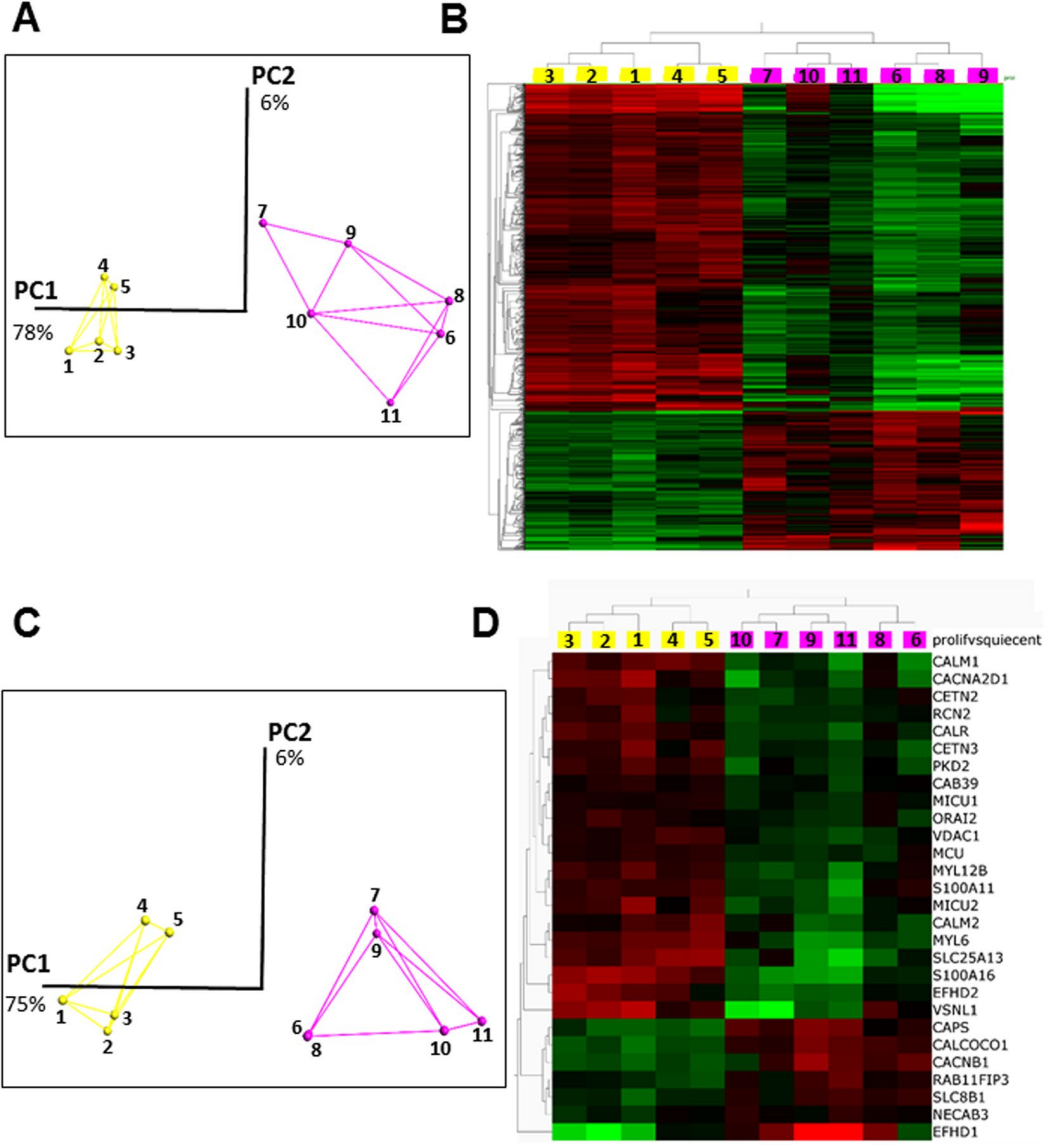
RNA signatures of proliferative and quiescent GSLCs. RNAS-seq data were analyzed using Qlucore software as described under materials and methods. We used a q-value of 5% to retain 1419 genes differentially expressed between proliferative and quiescent TG1 and TG1-C1 cells (see supplemental materials for the list of the 1419 genes). Yellow color is used for proliferative cells and pink color for quiescent cells. (**A**) Three-dimensional representation of principal component analyses for the 1419 genes significantly and differentially expressed between the proliferative and the quiescent GSLCs (for culture conditions see below and Table S1). The two principal components represent 78% and 9% of the information respectively. (**B**) Heat-map of differential gene expression for the 1419 selected genes. Each column represents the different experimental conditions (see below and table S1) and each lines represents a single gene. Expression levels are colored green for low intensities and red for high intensities. (**C**) Three-dimensional representation of principal component analyses for the 28 genes of the Ca^2+^ toolbox significantly and differentially expressed between the proliferative and the quiescent GSLCs. The link between experiments illustrates a proximity. The two main components represent 79% and 8% of the information, respectively. (**D**) Heat-map using the same set of 28 genes analyzed in Fig. 2C. Culture conditions are as followed: For proliferative conditions; Experiments #1–3, TG1 cells in NS34 medium at pH 7.4; Experiments # 4–5, TG1_C1 cells in NS34 medium at pH 7.4. For quiescent conditions; Experiments #6–7; no replacement of NS34 medium during 9 days for TG1 and TG1_C1 cells respectively; Experiments #8–9, NS34 pH 6.2 during 5 days for TG1 and TG1_C1 cells respectively; Experiment #10, NS34 pH 6.5 during 5 days for TG1 cells and Experiment #11, SKF96365 (10 µM) in NS34 pH 7.4 for TG1 cells.

To characterize the transcriptional changes occurring during quiescence, we performed RNA-sequencing (RNAseq) of TG1 and TG1_C1 cells grown under proliferating conditions and induced to quiescence by either non-renewal of the medium during 9 days or by acidification of pH_e_ to pH 6.5 or pH 6.2 (Supplementary Table S1). RNASeq processed gene data were variance filtered using a threshold of 0.11. A two-group comparison (proliferative versus quiescent cells) was then used to detect genes differentially expressed. We identified a group of 1419 genes using a q-value (the adjusted p-value found using an optimised FDR approach) of 5% to retain the genes with the most significant differential expression (Fig. 2). Principal component analysis (PCA) was used to visualize the variability of our samples. The first axis (PC1) collects 78% of the variance and clearly distinguishes proliferative and quiescent cells, whatever the origin of the samples or the treatment to obtain quiescence (Fig. 2A). Furthermore, the entire set of proliferative samples clustered, pointing to a great similarity between them whatever GSLC clones used (TG1 or TG1_C1). The slightly larger dispersion of the clusters for quiescent cells (Fig. 2A) is certainly related to the difference in the conditions used to reach quiescence. Of note, the group of down-regulated genes in the quiescent samples includes most of the genes belonging to the quiescent gene signature as described for adult stem cells^6^. Bioinformatics analysis of the gene enrichment for the selected set of genes discloses three main pathways, namely regulation of cell cycle, spindle assembly checkpoint and E2F target genes (Fig. 2B).

Since the remodeling of Ca^2+^ signaling contributes to cancer hallmarks such as excessive proliferation, survival or resistance to cell death^18^, we focused on genes belonging to the Ca^2+^ signaling toolkit^19^. A set of 105 genes expressed in GSLCs cells were unveiled (Supplementary Table S2). PCA confirmed the clustering of the proliferative GSLCs on the one hand and the quiescent GSLCs on the other hand (Fig. 2C). Using the analysis strategy described above, with the same parameters, a group of 28 genes differentially expressed was further selected (Fig. 2D) suggesting that Ca^2+^ genes recapitulate the information necessary to distinguish proliferative and quiescent states of the GSLCs. Ca^2+^ signalling is characterized by its spatiotemporal dynamics resulting from the interplay between Ca^2+^ fluxes and Ca^2+^ release and/or uptake by intracellular organelles^20^ and the mitochondrial Ca^2+^ uptake capacity contributes also to the shaping of the Ca^2+^ signal itself^21^. Importantly, we found that some genes associated with the regulation of Ca^2+^ influx through plasma membrane Ca^2+^ channels are up-regulated (*CACNB1*, *CAPS*) or down-regulated (*CACNA2D1*, *PKD2*, *ORAI2*) under quiescent conditions. Moreover, the mitochondrial Ca^2+^ uniporter (*MCU*), two of its modulators (*MICU1* and *MICU2*) and the voltage-dependent anion channel of the outer mitochondrial membrane (*VDAC1*), responsible for the transfer of Ca^2+^ from the endoplasmic reticulum (ER) to the mitochondria were down-regulated in quiescent cells whereas the mitochondrial Na^+^/Ca^2+^ exchanger (*SLC8B1*) was up-regulated (Fig. 2D). This suggests that quiescence is associated with changes in Ca^2+^ homeostasis through regulation of Ca^2+^ influx and of mitochondrial Ca^2+^ uptake.

### Remodelling of SOCE activity during the transition from proliferation to quiescence

**Figure 3:**
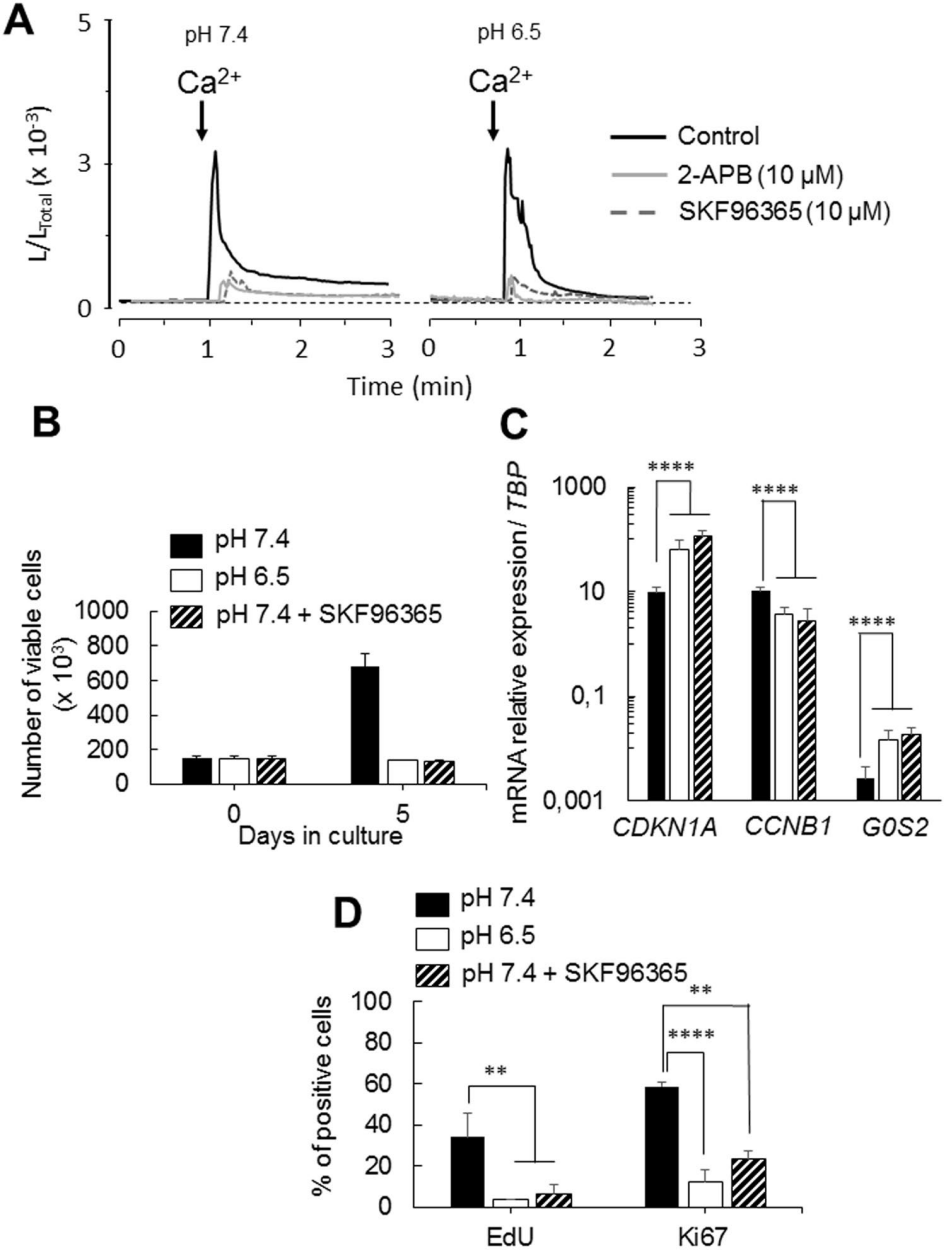
Remodeling of SOCE activity between proliferative and quiescent GSLCs. (**A**) Representative photo-multiplier (PMT) traces obtained from CytGA expressing TG1 cells in proliferating (left panel, pH 7.4) or in quiescent (right panel, pH 6.5) conditions (black traces) or in presence of 10 µM of inhibitors of SOC channels; SKF96365 (dotted traces) or 2-APB (grey traces). Values are plotted as L/L_TOTAL_ and every trace is the mean of 3 independent experiments. Prior to recording cells were washed with Ca^2+^-free medium, the arrows indicate the time at which medium containing 1 mM Ca^2+^ is perfused. (**B**) Cell proliferation measured by counting the number of viable cells over 5 days in NS34 at pH 7.4 in absence (black bars) or presence of SKF96365 (10 µM) (striped bars) and in NS34 at pH 6.5 (open bars). Each measure was done in triplicates with 3 independent experiments. (**C**) Expression of *CDKN1A*, *CCNB1* and *G0S2* was assessed by QRT-PCR in TG1 cells after 5 days in NS34 at pH7.4 in absence (black bars) or presence of SKF96365 (10 µM) (striped bars) and in NS34 at pH 6.5 (open bars). Results are given relative to *TBP* (TATA-Box Binding Protein) expression level. Error bars are derived from 11 independent experiments. (**D**) Histogram plot of the percentage of EdU and Ki67 positive cells in NS34 at pH 7.4 in absence (black bars) or presence of SKF96365 (10 µM) (striped bars) and in NS34 at pH 6.5 (open bars), determined by analysis of 6 confocal microscopy fields. Error bars are derived from 3 independent experiments.

Store-operated Ca^2+^ entry (SOCE) is a major mechanism of Ca^2+^ entry in non-excitable cells. SOCE controls cell proliferation in numerous cell types including cancer cells^22–24^. To examine SOCE between proliferating and quiescent TG1 cells, Ca^2+^ measurements were performed using EGFP-Aequorin targeted to the cytosol (CytGA) (Supplementary Fig. S4). While in proliferating TG1 cells SOCE is characterized by a transient Ca^2+^ rise followed by a sustained plateau, quiescent cells displayed a transient Ca^2+^ increase with a rapid decay and no plateau phase (Fig. 3A). Similar results are obtained with the TG1_C1 cells (Supplementary Fig. S4C). SKF96365 (10 µM), a pharmacological compound known to have inhibitory effects on Ca^2+^ influx through SOC^25^, significantly decreased the SOC-mediated Ca^2+^ transients in both proliferating and quiescent TG1 cells (Fig. 3A). Similar results were obtained with 2-APB (10 µM), another inhibitor of SOCE^25^. The change in the kinetics of the SOCE decay phase in quiescent GSLCs might reflect modifications in the mechanism of Ca^2+^ re-uptake in these cells.

Ca^2+^ is known to control the cell-cycle^26,27^. TG1 cells were therefore cultured in proliferating medium during 5 days in presence of the membrane-permeant Ca^2+^ chelator EGTA-AM (5 or 10 µM) to inhibit intracellular Ca^2+^ increase. Although this treatment blocks proliferation (Fig. S5A), it was not able to induce quiescence as shown by the mRNA level of expression for CDKN1A, CCNB1 and G0S2 (Supplementary Fig. S5B). These results were reinforced by the mRNA expression levels of other cyclins; *CCND1*, *D3*, *E2* and *A2*, of PCNA (Proliferating Cell Nuclear Antigen)^28^ and of *Hes1*. CCND1 and CCND3 are required during G1, CCNE2 for the G1/S transition and CCNA2 for the S/G2 transition^29^, Hes1 is a transcription factor involved in the control of the reversibility of cellular quiescence^30^. Figure S5C clearly indicates that EGTA-treated GSCLs display a mRNA expression profile similar to the one of pH 7.4-cultured GSCLs. In addition, 65.2% + 4.4% (n = 5 independent experiments) of the EGTA-treated TG1 cells still expressed the proliferative marker Ki67 (Fig. S5D). Altogether these data indicate that EGTA-treated GSCLs are arrested in cell-cycle but not in G0. Interestingly, the inhibition of Ca^2+^ influx through SOC by SKF96365 (10 µM) triggers quiescence of TG1 cells in proliferating medium (Fig. 3B–D and Supplementary Fig. S5E). Indeed, the inhibition of SOC channels with SKF96365 significantly decreased cell proliferation (n = 3, p < 0.0001) and is associated with the up-regulation of *CDKN1A* and *G0S2* and the down-regulation *CCNB1* similarly to quiescent GSLCs cultured in acidic pH_e_ medium (Fig. 3B,C). Similar results were obtained with the BTIC25 cells (Fig. S3B,C). The RNAseq analysis confirmed that cells in which Ca^2+^ influx through SOC was inhibited exhibit a quiescent transcriptomic signature (Fig. 2). Principal component analyses (Fig. 2A,C) showed that SKF96365-treated TG1 cells (experiment #11, Table S1) clearly clustered with the groups of TG1 cells where quiescence was induced by lowering pH_e_. Altogether, these data indicate that the inhibition of the SOCE not only induces cell-cycle arrest but triggers TG1 cells to adopt a quiescent state and suggests that the transition from proliferation to quiescence involves the remodelling of Ca^2+^ signalling.

SOCE is mediated by ORAI channels, located at the plasma membrane and STIM1, an ER membrane protein^31^. Although RNAseq data points to a down-regulation of *ORAI2* in all quiescent conditions (Supplementary Table S2), RT-QPCR analysis of the mRNA level for *STIM1* and its homologue *STIM2* or the *ORAI1* and *ORAI2* family members showed no significant difference between proliferating and quiescent cells but *ORAI3* expression increased significantly in TG1 cells induced to quiescence by lowering pH_e_ to 6.5 (Supplementary Fig. S6). This suggests that the difference in SOCE signals observed might not only be due to differential transcriptional regulations of *ORAI* and *STIM* family members.

**Figure 4:**
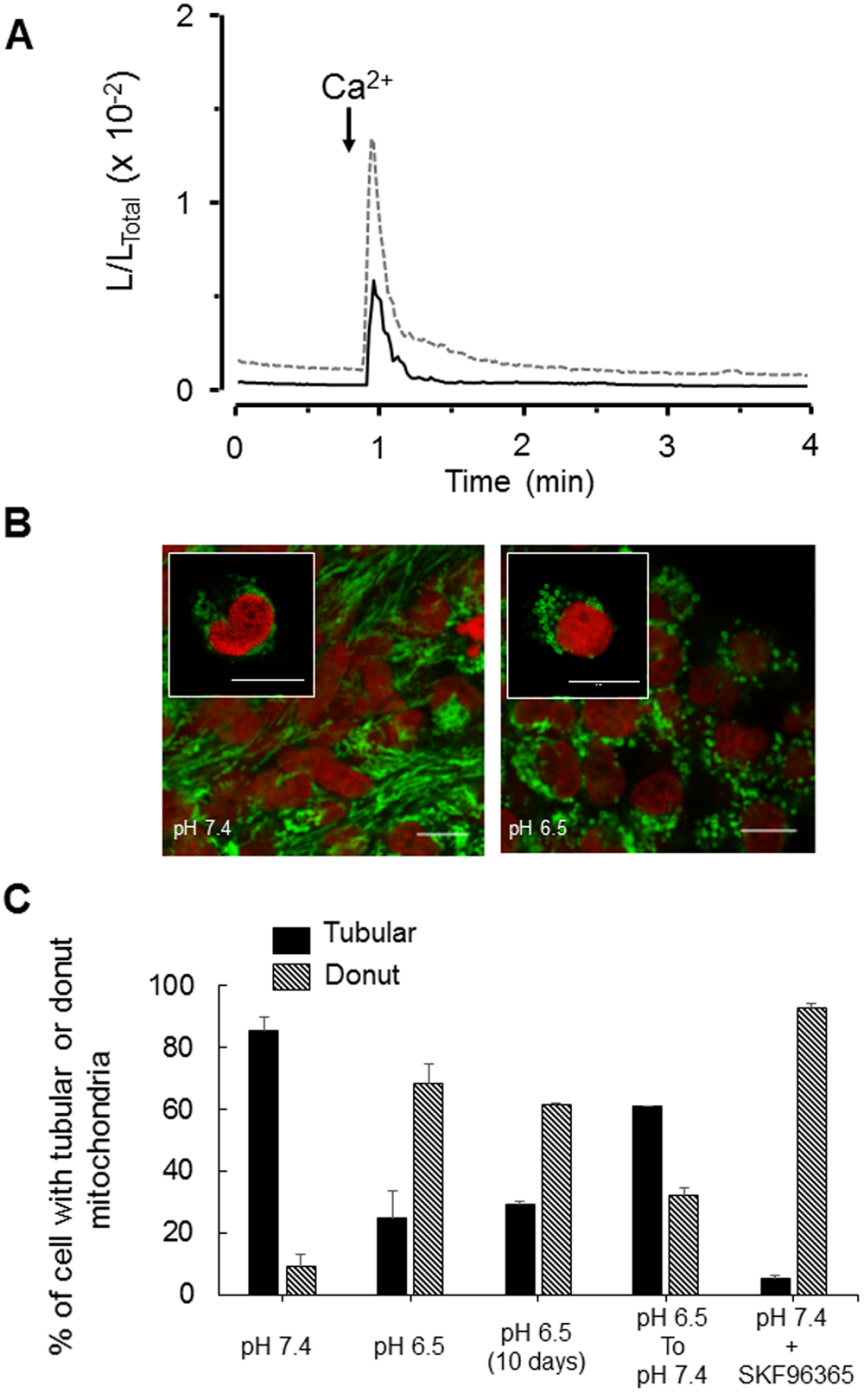
Remodeling of mitochondrial Ca^2+^ uptake and mitochondrial shapes in proliferative and quiescent GSLCs. (**A**) Representative PMT traces obtained from MitGA expressing TG1 cells in proliferating (black line, pH 7.4) or in quiescent (dotted grey line, pH 6.5) conditions. Values are plotted as L/L_TOTAL_ and every trace is the mean of 3 independent experiments. Prior to recording cells were washed with Ca^2+^-free medium, the arrows indicate the time at which medium containing 1 mM Ca^2+^ is perfused. (**B**) Confocal microscopy analysis of mitochondria shapes in proliferating (left panel, pH 7.4) and in quiescent TG1 cells (right panel, pH 6.5). Mitochondria were labeled with TOM20 antibody (green) and nucleus with Draq5 (red). Tubular mitochondria are found in proliferating TG1 cells (left panel and Inset) and donut-shaped mitochondria in quiescent TG1 cells (right panel and Inset). (**C**) Tubular (filled black bars) and donut-shaped mitochondria (striped black bars) quantified for the different culture conditions as indicated. Pictures were taken with a 63X 1.40 N.A. objective on a Leica SP8 upright confocal microscope. Scale bars: 10 µm in Insets; 5 µm for the other pictures.

We hypothesized that the difference in Ca^2+^ kinetics might be due to the efficiency of Ca^2+^ recapture. Mitochondria contribute to the regulation of Ca^2+^ homeostasis by controlling SOCE; this function occurs via mitochondrial Ca^2+^ uptake^32,33^. We compared Ca^2+^ uptake by mitochondria in proliferating and quiescent TG1 and TG1_C1 cells using the bioluminescent Ca^2+^ reporter EGFP-Aequorin targeted to the mitochondria (MitGA) (Supplementary Fig. S4) and observed that Ca^2+^ uptake in proliferating and quiescent GSLCs display different kinetics characterized by significantly higher amplitude in quiescent GSLCs (Fig. 4A). Altogether, these data suggest that in quiescent cells, Ca^2+^ entering through SOC channels is more efficiently recaptured, due to an increased activity of the mitochondrial Ca^2+^ uptake rather than through modulation of the SOCE mechanism itself.

### Mitochondrial dynamics in proliferating and quiescent TG1 cells

In glioma tumours, CSLCs are characterized by fragmented mitochondria relative to mitochondria from differentiated cancer cells^34^. We compared the mitochondrial morphology of proliferating and quiescent TG1 cells using the TOM20 antibody. Mitochondria of TG1 cells cultured at pH 7.4 display mostly a tubular shape, while a striking change in mitochondrial morphology was observed when TG1 cells are cultured for 5 days at pH 6.5, with the formation of donut-shaped mitochondria. Donut-shaped mitochondria were identifiable as circular structures with a central hole and a diameter of >0.8 µm (Fig. 4B). Indeed, while only 9% (n = 390, 4 independent experiments) of the proliferating TG1 cells have donut-shaped mitochondria, this proportion is increased in quiescent TG1 cells with 68% (n = 389, 4 independent experiments) showing donut-shaped mitochondria (Fig. 4C). This quiescence linked transition from tubular to donut- shaped mitochondria is also observed for BITC25 cells (Fig. S7). Interestingly, the reshaping of mitochondria is reversible, the percentage of donut-shaped mitochondria decreased when quiescent TG1 cells are transferred back to pH 7.4 (Fig. 4C). Since SKF96365-treated TG1 cells are quiescent, we next examined whether it is associated with the modification of mitochondrial morphology. 93% of the SKF96365-treated TG1 cells (n = 100, 3 independent experiments) have donut-shaped mitochondria (Fig. 4C). These observations suggest that donut-shaped mitochondria might be a characteristic feature of quiescent GSLCs.

### Mitochondrial morphology remodelling in glioblastoma tumorospheres

**Figure 5:**
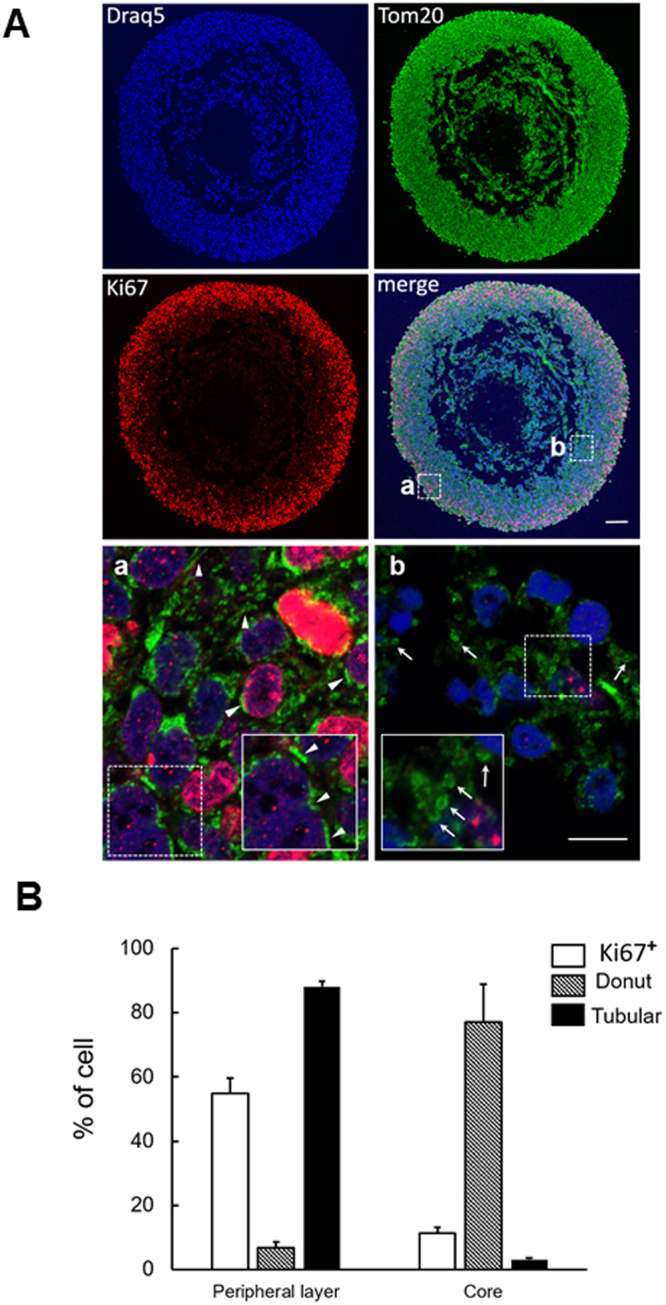
Mitochondrial morphology remodeling in glioblastoma tumorospheres. (**A**) Confocal microscopy analysis of TG1 macro-tumorosphere section after 8 weeks in culture. Proliferating cells were labeled with the Ki67 antibody (red), mitochondria morphology revealed with the TOM20 antibody (green) and nucleus stained with Draq5 (blue). Arrow-heads point to typical tubular-shaped mitochondria and arrows to typical donut-shaped mitochondria. Insets are enlarged images of selected areas (dotted squares) in (a) and (b) domains of the macro-tumorosphere. Pictures taken with a 10 × 0.30 N.A. objective for whole views of tumorospheres and with a 63X 1.40 N.A. objective for (a) and (b) details on a Leica SP8 upright confocal microscope. Scale bars: 100 µm in all images in A and 5 µm in Aa and Ab. (**B**) Histogram plot of the number of cells with at least one tubular-shaped mitochondria or one donut-shaped mitochondria in the outer rim (a) and in the core (b) domains of the tumorosphere. 6 areas were counted in each domain.

*In vitro*, GSLCs can form large floating spheres of more than 500 µm diameter, called macro-tumorospheres^35^ which present strong similarities in their cellular organization with the 3D organoid culture systems or glioblastoma tumours *in vivo*^5,36^. We previously showed that TG1 or TG1_C1 GSLCs form such macro-tumorospheres characterized by cellular heterogeneity in terms of structure and cell composition with proliferating Ki67 positive cells localized to the outer rim while slow-cycling cells are found in the central hypoxic, necrotic and probably acidic core^14^. These macro-tumorospheres constitute a favourable *in vitro* assay to compare mitochondria morphology. To this end, mitochondria were labelled with the TOM20 antibody (Fig. 5Aa,b). Quantification as detailed in the method section, revealed significantly higher number of cells with donut-shaped mitochondria (77%, 3 independent experiments) in the core as compared to the periphery (7%, 3 independent experiments) (Fig. 5B). These data confirm the observation obtained with isolated TG1 cells and show that within the macro-tumorospheres, the mitochondrial morphology undergoes remodelling according to the proliferating status of the GSLCs.

## Discussion

Glioblastoma tumour recurrence has been attributed to the GSLCs which reside within the tumour mass in hypoxic and acidic microenvironments in a slow-cycling or quiescent state. However, standard *in vitro* culture conditions which favour proliferating GSLCs over quiescent ones fail to reproduce the tumour conditions and preclude studies on the mechanisms controlling quiescence. We established that modification of the culture condition by lowering pH_e_ from pH 7.4 to pH 6.5 induces cell-cycle arrest in GSLCs as confirmed by the up-regulation of the expression of *CDKN1A* and *G0S2* and the down-regulation of *CCNB1*, without modification of their stemness properties as seen by the expression of the stemness markers, NANOG, OLIG2 and SOX2,. This cell-cycle arrest is reversible; GSLCs transferred back to proliferating culture condition at pH 7.4 rapidly resumed proliferation. Altogether these data indicate that the acidic treatment of GSLCs induces a quiescent G_0_ state.

This simple culture protocol allowed us to compare the transcriptomic profile of proliferating and quiescent GSLCs and to perform Ca^2+^ imaging to directly test their respective Ca^2+^ responses. Accumulating evidences suggest that Ca^2+^ might be an important regulator of tumorigenesis in GBM^37,38^. We found that in GSLCs maintained in acidic medium, genes associated with cell-cycle progression are down-regulated and that genes coding proteins involved in Ca^2+^ signalling, particularly plasma membrane and organelles Ca^2+^ channels, are modulated. We showed that the inhibition of SOC channels by SKF96365 not only blocks cell proliferation but was sufficient to lead GSLCs toward a quiescent state. This suggests that Ca^2+^ signalling through SOC channels is necessary to regulate the balance proliferation/quiescence. To the best of our knowledge, this is the first time that a causal role can be ascribed to Ca^2+^ signalling in the transition of cells from proliferation to quiescence. How is Ca^2+^ homeostasis regulated in this process? STIM1 and ORAI1 proteins have been identified as critical components for Ca^2+^ entry through SOC however, increasing evidence showed that alteration of STIM1 and ORAI1 but also of ORAI3 expression and function contribute to tumorigenesis^38,39^. Using aequorin-based Ca^2+^ recordings to follow intracellular Ca^2+^ fluxes, we observed that GSLCs cultured in stem-cell medium under proliferative or quiescent conditions have distinct kinetics of Ca^2+^ entry through SOC channels. This difference in kinetics could be due to differential expression of the genes coding for the STIM1/2 and/or ORAI1-3 family members. Here we showed that only the expression of *ORAI3* is modified between proliferating and quiescent GSLCs. Careful examination of the SOC kinetics revealed that the initial rate of Ca^2+^ influx is not modified in quiescent GSLCs but that the prolonged plateau is markedly attenuated. This difference in the SOC Ca^2+^ kinetics might rather be due to an increased efficiency of intracellular Ca^2+^ re-capture by quiescent GSLCs.

Mitochondria are crucial organelles which not only regulate cellular energy generation and apoptosis but also control intracellular Ca^2+^ signalling particularly by their capacity to take up Ca^2+^ thereby modulating SOC channels^40^. This capacity of mitochondria to actively buffer cytosolic Ca^2+^ occurs via the mitochondrial Ca^2+^ uniporter MCU associated with regulatory subunits including MICU1, MICU2 and EMRE^41^. Our data on Ca^2+^ homeostasis with the bioluminescent probe Aequorin support this view. We observed that the stimulation of Ca^2+^ entry via SOC channels by depletion of the internal ER stores triggered a large mitochondrial Ca^2+^ transients in GSLCs induced to quiescent by lowering pH_e_ to 6.5 compared to proliferative GSLCs at pH 7.4. While these results would suggest that the activity of MCU is enhanced in quiescent GSLCs, our RNAseq data point to a reduced expression of MCU and of two of its regulators, MICU1 and MICU2 in quiescent GSLCs compared to proliferative GSLCs. It has been shown in the literature that the rate of Ca^2+^ entry through MCU depends on the cytosolic Ca^2+^ concentrations ([Ca^2+^]_cyt_), with slow rate for low [Ca^2+^]_cyt_ and faster rate when the [Ca^2+^]_cyt_ increases. This sigmoidal behaviour has been mainly attributed to the functions of MICU1 and MICU2. Although their respective gatekeeping functions are still debated, experimental evidences clearly indicate that MICU2 silencing induces an increase of mitochondrial Ca^2+^ uptake, indicating that MICU2 is an inhibitor of MCU. On the contrary, MICU1 behaves as a Ca^2+^-sensitive switch for MCU activity; acting as inhibitor of MCU at low [Ca^2+^]_cyt_ and activator at high [Ca^2+^]_cyt_^42^. We observed that the resting level of cytosolic Ca^2+^ is lower in proliferating TG1 cells compared to quiescent ones. In proliferating TG1 cells, MICU1 and MICU2 should therefore be inhibitors of MCU and its activity should be low. However, in quiescent TG1 cells we found that MICU2 expression was reduced and as a consequence, its inhibitory effect on MCU released. Although MCU expression is reduced in quiescent cells, MCU activity would be enhanced. Our RNAseq data also pointed to the overexpression, in quiescent cells, of the Na/Ca^2+^ exchanger *SLC8B1*, recently identified as one of the components responsible for mitochondrial Ca^2+^ efflux and which counteracted the accumulation of Ca^2+^ into mitochondrial matrix by MCU^43^. Therefore, the transcriptional remodelling of components that control mitochondrial Ca^2+^ homeostasis may contribute to prevent mitochondrial Ca^2+^ overload during quiescence.

It has been reported that mitochondria are highly dynamic organelles whose morphology is controlled by fission-fusion mechanisms. Under normal conditions mitochondria are essentially tubular but can be fragmented during apoptosis or formed distended swollen structures during necrosis^44^. Besides these tubular or fragmented shapes, a third shape called donut was reported during hypoxia-reoxygenation stress^45^. Donut-shaped mitochondria result from the bending and the fusion of the two ends of tubular mitochondria^46^. We observed that proliferative GSLCs have mainly tubular mitochondria, while quiescent GSLCs mainly show donut-shaped mitochondria. This reshaping of mitochondria is reversible. When GSLCs are transferred from a quiescent to a proliferative medium donut-shaped mitochondria switch back to a tubular shape. Recent studies on the relationship between mitochondrial shapes and stress show that the transition from tubular to donut requires an increase of mitochondrial Ca^2+^ uptake^47^ and does not involve fusion mechanism^45^. Our results are in accordance with the requirement of enhanced mitochondrial Ca^2+^ uptake and the formation of donut-shaped mitochondria. This remodelling of mitochondrial morphology from tubular to donut may be an additional mechanism explaining the increased mitochondrial Ca^2+^ uptake capacity observed after SOC stimulation in quiescent GSLCs. An interesting observation that may contribute to the formation of donut-shaped mitochondria during quiescence is the down-regulation of the *LETM1* gene (see RNAseq data). LETM1 is a leucine zipper and EF-hand transmembrane protein localized to the inner mitochondrial membrane^48^ which has been shown to be required to maintain the mitochondrial tubular network independently of the mechanism of mitochondrial fission which involved the DRP1-dependent pathway^49^.

Mitochondrial dysfunction is one of the hallmarks of cancers and this is associated with cancer cells exhibiting fragmented mitochondria^50^. On large macro-tumorospheres, we found that a large fraction of mitochondria forms a tubular network in proliferating GSLCs while GSLCs localized to the core of the tumorosphere are characterized by donut-shaped mitochondria. Importantly, the presence of a hypoxic environment has been documented within the core of these macro-tumorospheres^14^. As suggested by^45^, the formation of donut-shaped mitochondria might represent a protective mechanism preserving mitochondria against autophagy upon metabolic stress.

In conclusion, our findings on mitochondria in GSLCs point out the regulation of mitochondria dynamics and ER network. The relationship between changes in mitochondrial shape and physiological parameters, particularly mitochondrial Ca^2+^, has therefore a great potential for translational research. Mitochondria targeted therapeutic may involve the control of the shape correlated with Ca^2+^ signalling.

## Methods

### Cells and growth conditions

GSLCs (TG1, TG1_C1 and BITC25) lines were isolated, established and cultures from surgical resections of primary GBMs from adult patients (age over 18) after informed consent as previously described^12,17,51^. These cells express stem cells markers, have clonal properties, were able to differentiate and induce tumours when orthotopically grafted in immune-compromised mice^12,52^. Their mutational status is however different, while BITC25 is mutated for TP53, TG1 and TG1_C1 are not^12,52^. The cells are expanded in NS34 serum-free medium containing EGF and bFGF (DMEM-F12 1/1, glutamine 10 mM, Hepes 10 mM, Sodium bicarbonate 0.025%, N2, G5, and B27) as described^53^ and used up to 10 passages. Under the culture conditions used, TG1, TG_C1 and BTIC25 cell grew as floating neurospheres. To induce quiescence, GSLCs were either cultured 9 days without medium removal as described^13^ or 5 days in NS34 medium buffered to pH 6.2 or 6.5. Macro-tumorospheres from single GSLCs were obtained as described in^14^.

### Cell proliferation, viability and EdU assay

Cells were seeded at 1∙10^5^cells/ml in 6-well plate and proliferation assessed by direct cell counting method using Neubauer Improved C-C-Chip. Cell viability was evaluated using Trypan blue exclusion assay. The number of cells in S phase was determined with Click-it® Edu Alexa fluor 647 Imaging Kit (Invitrogen) as indicated. At day 5, cells were incubated with 10 µM EdU solution during 2 hours, fixed in 16% paraformaldehyde (PFA) for 30 minutes, transferred on SuperFrost slides (Thermo Scientific, France) and observed with Leica Sp8 confocal microscope.

### Sphere formation assay and formation of clonal tumorospheres from single GSLCs *in vitro*

To assess the ability of TG1 cells to form new clones, spheres were mechanically dissociated into single cell suspension and seeded into 24-wells plates at 10^4^ cells per well in soft-agar (0.7% agarose) NS34 medium at pH 7.4 or pH 6.5. This allows to continuously follow sphere formation from individual isolated TG1 cells during 7 days. Pictures were taken with a Nikon Eclipse TS100 Microscope, with 40X/0.55 and 20X/0.40 objectives from day 1 to day 7. Macro-tumorospheres from single GSLCs were obtained as described in^14^.

### qRT-PCR

Relative expression levels of several genes (*CDKN1A, CCNB1, GOS2, CCND1, CCND3, CCNE2, CCNA2, PCNA, Hes1, STIM1, 2, ORAI1, 2, 3*, *MICU1, 2* and *MCU*) relative to *TBP* (TATA-Box Binding Protein), in proliferating and quiescent TG1 cells were assessed by real-time qRT–PCR. Total RNA was extracted with RNeasy® mini kit (Qiagen) and quantified in a NanoDrop apparatus. RNA (0.4 μg) was reverse transcribed using q-Script^TM^ cDNA SuperMix (Quanta, Biosciences). Amplifications of 8 ng of cDNA were performed in triplicate using Eva-Green® SuperMix (Bio Rad) in a 10 μl reaction mixture containing 500 nM primers (S4 Table), run on BioRad CFX96 thermocycler.

### RNAseq data and analysis

To establish RNA signatures of proliferative and quiescent GSLCs, the following strategy was used. Several experiments were performed in order to take into account different types of variabilities (Supplementary Table S1); (i). To address variability due to laboratories environments, experiments were done in two laboratories (Strasbourg and Toulouse) following identical protocols. The cells were obtained from the same master cell bank and the composition of the growing media was identical. (ii) For cellular variability, two cell lines TG1 and TG1_C1 were used. (iii) for variability in inducing quiescence, the switch to the quiescent state was obtained by either the non-replacement of the medium during 9 days, or acidification of the medium to pH 6.5 or 6.2 for 5 days or treatment of the cells by 10 µM SKF96365 in the growing medium (pH 7.4) at day 1 and 3 and analysis at day 5. In normal medium at pH 7.4, the cells are in a proliferative state. Total RNA was extracted as described above and RNA quality was controlled with AATI Fragment Analyser (Advanced Analytical Technologies, Inc). RNA-seq was performed at the Strasbourg IGBMC platform. Short reads were aligned using the reference genome hg38 (http://genomeast.igbmc.fr). The Qlucore software (htpp://www.glucore.org) used for the analysis. Reads (fastq files) were mapped onto the hg38 assembly of the Homo sapiens genome using Tophat 2.0.10^54^ and bowtie version 2-2.1.0^55^ to give BAM files. From the BAM files and the hg38 GTF file, Qlucore Omics Explorer creates a count matrix by counting the number of reads overlapping each genomic feature in each sample (each BAM file). The counts are then normalized across samples and across genes, and finally log2-transformed. The processed data have also been deposited in the GEO datasets database under the accession number GSE 93991.

### Immunofluorescence Analysis

Proliferating or quiescent TG1 cells were cultured in suspension for 5 days at pH 7.4 and pH 6.5 respectively and processed as described^14^. Primary antibodies used in this study are listed in Supplementary Table S3.

### Analysis of mitochondrial morphology in clonal macro-tumorospheres

Sections of clonal macro-tumorospheres were obtained as described^14^. Mitochondria were labelled with an antibody against TOM20. For each section, 5 randomly selected fields were taken in the periphery and in the core. Cells containing at least one donut-shaped mitochondrion were counted as positive. Donut-shaped mitochondria were manually counted considering the structures with a central hole and a donut diameter >0.8 µm^46^.

### Gene construction

The chimeric GFP-aequorin (GA) targeted to the cytosol (CytGA) is a generous gift from Ph. Brûlet^56^. We constructed the chimeric GA targeted to mitochondria (MitGA). The starting sequence of GA was obtained by PCR from CytGA construct with primers containing a BamHI site at the 5′ end (CC**ggatcc**AGCAAGGGCGAGGAGCTGTTC) and XbaI site at the 3′ end (CC**tctaga**TTAGGGGACAGCTCCACCG). The mitochondrial targeting sequence was fused in frame at the 5′ end with a 86 bp sequence of the human cytochrome c oxidase VIII subunit (COX VIII) isolated from pDsRed2-Mito by digestion with BamHI and XbaI from pDsred2-Mito.

### Ca^2+^ measurements using aequorin probes

Cells (2∙10^6^) were electroporated with 2 µg of DNA encoding either the Mitochondrial-targeted EGFP-Aequorin (MitGA) or Cytosolic-targeted EGFP-Aequorin (CytGA) probes, using AMAXA Kit L cell line nucleofactor (Lonza). The localization of the Ca^2+^ reporter to mitochondria was confirmed using an antibody directed against a mitochondrial protein, TOM20, a 20 kDa translocase of outer mitochondrial membranes. Mitochondrial or cytosolic Ca^2+^ measurements were performed at 37 °C and the temporal aequorin-generated data were acquired as described^14^. Ca^2+^ measurements were performed on isolated TG1 cells immobilized in agar. Ca^2+^ influx through SOCE was obtained by challenging the SOC channels through depletion, in Ca^2+^-free medium, of ER store with thapsigargin (1 µM), an inhibitor of the ER Ca^2+^-ATPase. Following this treatment, bath application of 1 mM Ca^2+^ restores Ca^2+^ influx through SOC^57^.

### Statistical analysis

The results are expressed as means + S.D. The statistical significance was evaluated using the Student’s t-test or one-way ANOVA with GraphPad Prism6 software. *p < 0.05–0.001, ****p < 0.0001

### Accession numbers

All sequencing data are available from the GEO database (accession number: GSE 93991).

## Electronic supplementary material

supplementary information
