# Supplementary material for: Quiescence status of glioblastoma stem-like cells involves remodelling of Ca2+ signalling and mitochondrial shape
Source: Sci Rep. 2018 Jun 27;8:9731. doi: 10.1038/s41598-018-28157-8 (PMC6021377; doi:10.1038/s41598-018-28157-8)

Quiescence status of glioblastoma stem-like cells involves remodelling of Ca^{2+} signalling and mitochondrial shape

Authors

Francisco J. Aulestia[1], Isabelle Néant[1], Jihu Dong[2], Jacques Haiech[2], Marie-Claude Kilhoffer[2], Marc Moreau[1], and Catherine Leclerc[1,*]

Supplementary tables

Table S1. Culture conditions for TG1 and TG1-C1 cells

Experiment #	*Cell line*	*Culture conditions*	*cellular state*
1	TG1	NS34, pH 7.4	
2	TG1	NS34, pH 7.4	
3	TG1	NS34, pH 7.4	Proliferative (self-renewal)
4	TG1_C1	NS34, pH 7.4	
5	TG1_C1	NS34, pH 7.4	
6	TG1	No replacement of NS34 medium during 9 days	
7	TG1_C1	No replacement of NS34 medium during 9 days	
8	TG1_C1	NS34 pH 6.2 during 5 days	Quiescence
9	TG1	NS34 pH 6.2 during 5 days	
10	TG1	NS34 pH 6.5 during 5 days	
11	TG1	SKF-96365 (10µM) in NS34, pH 7.4	

Table S2. List of Ca^{2+} toolbox genes expressed in GSLCs.

Among the 250 genes belonging to the Ca^{2+} toolkit, 107 genes are expressed in GSLCs either in the proliferative or quiescent conditions (see Table S1). A fold change < 1 corresponds to down-regulation in quiescent GSLCs (highlighted in blue). Fold change is computer from normalized log2 transformed data as described in the experimental procedures. For a given gene, fold change is 2^(mean value quiescent cells – mean value proliferative cells). CaBP, Calcium binding protein; TF, Transcription factor; ER/SR, Endoplasmic Reticulum/Sarcoplasmic Reticulum; ND, subcellular location not determined.

Gene Symbol	Gene ID	Fold change	Function	Subcellular Location
VSNL1	7447	0.606635332	CaBP	Cytosol / Membrane
S100A3	6274	0.623029709	CaBP	Cytosol / Nucleus
SLC25A13	10165	0.643405378	Ca^{2+} outflow	Mitochondria
S100B	6285	0.670835435	CaBP	Cytosol / Nucleus
SLC24A3	57419	0.741691947	Ca^{2+} outflow	Plasma membrane
EFCAB13	124989	0.771550298	CaBP	*ND*
RASGRP1	10125	0.776597738	CaBP	*ND*
EFHD2	79180	0.777477086	CaBP	*ND*
MCU	90550	0.779598892	Ca^{2+} outflow	Mitochondria
MICU2	221154	0.779701352	Ca^{2+} outflow	Mitochondria
ITPR2	3709	0.791205823	Ca entry	ER/SR
S100A16	140576	0.799640954	CaBP	Cytosol
MYL6	4637	0.803082585	CaBP	*ND*
CETN3	1070	0.806671381	CaBP	Centrosome
PKD2	5311	0.824052572	Ca entry	Plasma membrane
CACNA2D1	781	0.825506866	Ca entry	Plasma membrane
S100A10	6281	0.829648793	CaBP	Cytosol / Nucleus
CACNA1C	775	0.830254197	Ca entry	Plasma membrane
NUCB2	4925	0.838697314	CaBP / DNA binding	Cytosol
ATP2B2	491	0.844160676	Ca^{2+} outflow	Plasma membrane
CALM1	801	0.85573566	CaBP	Cytosol
RASEF	158158	0.862091482	CaBP	Cytosol
RCN2	5955	0.863518476	CaBP	ER
CETN2	1069	0.864145756	CaBP	Centrosome
CALM2	805	0.864191592	CaBP	Cytosol
LETM1	3954	0.87683773	Ca^{2+} outflow	Mitochondria
EFHC1	114327	0.877512217	CaBP	Cytosol
S100A4	6275	0.880372941	CaBP	Cytosol / Nucleus
ORAI2	80228	0.882773757	Ca entry	Plasma membrane
CALM3	808	0.889790297	CaBP	Cytosol
ITPR3	3710	0.89111346	Ca entry	ER/SR
STIM2	57620	0.891906142	Ca entry	ER/SR
S100A1	6271	0.894057453	CaBP	Cytosol / Nucleus
VDAC1	7416	0.89487958	channel	Mitochondria
MYL12B	103910	0.89888984	CaBP	cytosol

CALU	813	0.899016738	CaBP	ER
MYL12A	10627	0.904072046	CaBP	Cytosol
S100A11	6282	0.906369627	CaBP	Cytosol / Nucleus
CALR	811	0.907804906	CaBP	ER
CAB39	51719	0.908084214	CaBP	Cytosol
MICU1	10367	0.912965178	Ca^{2+} outflow	Mitochondria
MYL6B	140465	0.913026094	CaBP	Cytosol
TRPM8	79054	0.916101217	Ca entry	Plasma membrane
ATP2B1	490	0.916224957	Ca^{2+} outflow	Plasma membrane
NCALD	83988	0.91999191	CaBP	Cytosol
S100A2	6273	0.921293378	CaBP	Cytosol / Nucleus
MCTP1	79772	0.92139107	CaBP	Membrane
TPCN2	219931	0.922363162	Ca entry	Membrane / Lysosome
SLC25A12	8604	0.928004563	Ca^{2+} outflow	Mitochondria
SRI	6717	0.93116945	CaBP	Cytosol / SR
VDAC3	7419	0.931555867	channel	Mitochondria
CARHSP1	23589	0.940751493	CaBP	Cytosol
ATP2C1	27032	0.949672997	Ca^{2+} outflow	Plasma membrane
S100A13	6284	0.951274991	CaBP	Cytosol / Nucleus
S100A6	6277	0.953499198	CaBP	Cytosol / Nucleus
TRPM7	54822	0.956226528	Ca entry	Plasma membrane
EFCAB11	90141	0.958138525	CaBP	*ND*
ATP2B4	493	0.961005092	Ca^{2+} outflow	Plasma membrane
STC1	6781	0.964208543	CaBP	Secreted
ORAI1	84876	0.964939058	Ca entry	Plasma membrane
HPCAL1	3241	0.967616558	CaBP	Membrane
CAB39L	61617	0.971555114	CaBP	*ND*
MYL9	10398	0.972350121	CaBP	Cytosol
NCS1	23413	0.975012004	CaBP	Cytosol
MICU3	286097	0.978178382	Ca^{2+} outflow	Mitochondria
TRPC1	7220	0.988282204	Ca entry	Plasma membrane
VDAC2	7417	0.996251523	channel	Mitochondria
PSEN1	5663	0.999276936	CaBP	Membrane
GCA	25801	0.999806046	CaBP	Cytosol
CAPS2	84698	1.00509083	CaBP	*ND*
CACNG4	27092	1.00808823	Ca entry	Plasma membrane
ATP2A2	488	1.01196682	Ca^{2+} outflow	ER/SR
CACNB4	785	1.01560533	Ca entry	Plasma membrane
EFCAB14	9813	1.01783824	CaBP	*ND*
RCN1	5954	1.02335477	CaBP	ER
CHP1	11261	1.03763425	CaBP	Cytosol / Nucleus
NUCB1	4924	1.0463016	TF	Golgi
PSEN2	5664	1.05628753	CaBP	Membrane
CACFD1	11094	1.06460106	Ca entry	Plasma membrane
SLC24A1	9187	1.0790931	Ca^{2+} outflow	Plasma membrane
CLGN	1047	1.09286618	CaBP	ER

SLC25A23	79085	1.09671128	Ca^{2+} outflow	Mitochondria
CACNA1H	8912	1.10686553	Ca entry	Plasma membrane
CACNB3	784	1.11599135	Ca entry	Plasma membrane
ATP2A3	489	1.13170516	Ca^{2+} outflow	ER/SR
ATP2A1	487	1.13189495	Ca^{2+} outflow	ER/SR
PKD1L2	114780	1.13193941	Ca entry	Membrane
NECAB3	63941	1.13303959	CaBP	Golgi
STIM1	6786	1.13753235	Ca entry	ER/SR
CALCOCO2	10241	1.14259505	CaBP	Cytosol
ITPR1	3708	1.15209162	Ca entry	ER/SR
TRPM4	54795	1.19094431	Ca entry	Plasma membrane
RAB11FIP3	9727	1.19934535	CaBP	Cytosol / Centrosome
PKD1	5310	1.19998717	Ca entry	Plasma membrane
GUCA1B	2979	1.25257576	CaBP	Membrane
ZZEF1	23140	1.29527771	TF	*ND*
RCN3	57333	1.35777569	CaBP	ER
SLC8B1	80024	1.40965879	Ca entry	Mitochondria
CALCOCO1	57658	1.41236198	CaBP	Cytosol
ORAI3	93129	1.43146002	Ca entry	Plasma membrane
MYL5	4636	1.44176078	CaBP	Cytosol
CARF	79800	1.45379829	TF	Nucleus
TPCN1	53373	1.50876069	Ca entry	Membrane / Lysosome
CAPS	828	1.87185323	CaBP	Cytosol
EFHD1	80303	1.93805242	CaBP	Mitochondria
CACNB1	782	2.0890739	Ca entry	Plasma membrane

Table S3. List of antibodies used.

Name	***Type, Company***	***Dilution***
Anti-Sox2	Rabbit Polyclonal, *Abcam*	1/100
Anti-Nanog	Goat Polyclonal, *R&D Systems*	1/100
Anti-Olig2	Rabbit Polyclonal, *Millipore Bioscience*	1/500
Anti-TOM20	Rabbit Polyclonal, *Santa Cruz Bioechnology*	1/100
Anti-Ki67	Mouse, *Dako*	1/100
Anti-GFP	Mouse, *Roche*	1/1000

Table S4. Sequences of primers used

Gene name	Accession number	Primer sequences (5'-3')	Position	Amplicon size	References
Tata box BP	NM_003194	GAGCTGTGATGTGAAGTTTCC	1059	117 bp	(1)
		TCTGGGTTTGATCATTCTGTAG	1176		
CDKN1A (p21)	NM_000389	GACTCTCAGGGTCGAAAACG	531	94 bp	(2)
		GGATTAGGGCTTCCTCTTGG	623		
CCNB1	NM_031966	TCTTCCAGTTATGCAGCACCT	1381	103 bp	primer3
		AGCATGCTTCGATGTGGCAT	1464		
G0S2	NM_015714.3	AAGGGGAAGATGGTGAAGCTG	315	85 BP	primer3
		CTGCACACAGTCTCCATCAGG	400		
CCNA2	NM_001237	GAAGATGAAAAGCCAGTGAGTGT	798	120 bp	primer3
		TGGCTGTTTCTTCATGTAACCC	896		
CCND1	NM_053056.2	CCTGTCCTACTACCGCCTCA	878	59 bp	(3)
		CAGTCCGGGTCACACTTGA	937		
CCND3	NM_001760	AGGGATCACTGGCACTGAAGT	909	102 bp	Primer3
		CTGGAGCTGGTCTGAGAGGC	971		
CCNE2	NM_057749	TTTGGCTATGCTGGAGGAAGT	1193	103 bp	Primer3
		AGTGCTCTTCGGTGGTGTCA	1276		
HES1	NM_005524.3	ACGTGCGAGGGCGTTAATAC	618	89 bp	(4)
		GGGGTAGGTCATGGCATTGA	707		
PCNA	NM_002592	GCCACTCCACTCTCTTCAACG	911	120 bp	Primer3
		ATCCTCGATCTTGGGAGCCA	991		
STIM1	NM_003156.3	TGTGGAGCTGCCTCAGTATG	1000	108 bp	(5)
		CTTCAGCACAGTCCCTGTCA	1108		
STIM2	NM_020860	GACGTCAGTATGCAGAACAG	1478	87 bp	(5)
		GACCAACTGCTTCTCAGTTC	1565		
ORAI1	NM_032790	ATGGTGGCAATGGTGGAG	494	121 bp	(5)
		CTGATCATGAGCGCAAACAG	615		
ORAI2	NM_032831	GCAGCTACCTGGAACTGGTC	349	175 bp	(6)
		CGGGTACTGGTACTGCGTCT	524		
ORAI3	NM_152288	GGCCAAGCTCAAAGCTTCC	380	104 bp	(7)
		CCTGGTGGGTACTCGTGGT	484		
MCU	NM_138357.2	CGCCAGGAATATGTTTATCCA	877	162 bp	primer3
		CTTGTAATGGGTCTCTCAGTCTCTT	1039		
CCDC109B	NM_017918	GGCCTTCCCTTGGTAACACT	338	156 bp	(8)
		GTTGCCATCTGCTGTGAAGA	493		
MICU1	NM_006077.3	ACAGTGGCTAAAGTGGAGC	1393	222 bp	primer3
		GTTTGGGTAAAGCGAAGTCC	1615		
MICU2	NM_152726	GGCAGTTTTACAGTCTCCGC	255	153 bp	(9)
		AAGAGGAAGTCTCGTGGTGTC	400		

1. Valente V*, et al.* (2009) Selection of suitable housekeeping genes for expression analysis in glioblastoma using quantitative RT-PCR. *BMC Mol Biol* 10:17.
2. Saigusa S*, et al.* (2012) Gene expression profiles of tumor regression grade in locally advanced rectal cancer after neoadjuvant chemoradiotherapy. *Oncol Rep* 28(3):855-861.
3. Tobin NP, Sims AH, Lundgren KL, Lehn S, & Landberg G (2011) Cyclin D1, Id1 and EMT in breast cancer. *BMC Cancer* 11:417.

4. Gao F, *et al.* (2014) Hes1 is involved in the self-renewal and tumourigenicity of stem-like cancer cells in colon cancer. *Sci Rep* 4:3963.
5. El Boustany C, *et al.* (2010) Differential roles of STIM1, STIM2 and Orai1 in the control of cell proliferation and SOCE amplitude in HEK293 cells. *Cell Calcium* 47(4):350-359.
6. Motiani RK, Abdullaev IF, & Trebak M (2010) A novel native store-operated calcium channel encoded by Orai3: selective requirement of Orai3 versus Orai1 in estrogen receptor-positive versus estrogen receptor-negative breast cancer cells. *J Biol Chem* 285(25):19173-19183.
7. Borowiec AS, *et al.* (2014) Are Orai1 and Orai3 channels more important than calcium influx for cell proliferation? *Biochim Biophys Acta* 1843(2):464-472.
8. Raffaello A, *et al.* (2013) The mitochondrial calcium uniporter is a multimer that can include a dominant-negative pore-forming subunit. *EMBO J* 32(17):2362-2376.
9. Patron M, *et al.* (2014) MICU1 and MICU2 finely tune the mitochondrial Ca2+ uniporter by exerting opposite effects on MCU activity. *Mol Cell* 53(5):726-737.

Quiescence status of glioblastoma stem-like cells involves remodelling of Ca^{2+} signalling and mitochondrial shape

Authors

Francisco J. Aulestia[1], Isabelle Néant[1], Jihu Dong[2], Jacques Haiech[2], Marie-Claude Kilhoffer[2], Marc Moreau[1], and Catherine Leclerc[1,*]

Supplementary Legends

Figure S1. In vitro induction of quiescence in TG1 cells.

(A) Trypan blue viability test. TG1 cells at pH7.4 (filled black bars) and pH 6.5 (striped black bars) and TG1_C1 cells at pH7.4 (filled grey bars) and at pH6.5 (striped grey bars). Each measure was done in triplicates and with 3 independent experiments. **(B)** Histogram plot of the percentage of EdU and Ki67 positive cells in NS34 at pH 7.4 (black bars) or pH 6.5 (white bars), determined by analysis of 6 confocal microscopy fields. Error bars are derived from 3 independent experiments. **(C)** TG1 cells induced to quiescence continue to express stemness markers. Immunofluorescence analysis of stemness markers (NANOG, OLIG2 and SOX2) after 5 days in culture at pH 7.4 (proliferating condition, left panel) and at pH 6.5 (quiescent condition, right panel). Nuclei were visualized by Draq5 staining. Pictures taken with a 63X 1.40 N.A. objective on a Leica SP8 upright confocal microscope. Scale bar = 20 µm.

Figure S2. The cell-cycle arrest induced by low extracellular pH is reversible.

(A) Proliferating TG1 cells were cultured for 5 days in NS34 medium at pH 6.5 to induce quiescence and then either transferred back to NS34 at pH 7.4 (black dots) or to NS34 at pH 6.5 (grey squares) for an additional 5 days and the number of viable cells counted. **(B)** Histogram plot of the percentage of EdU and Ki67 positive cells in NS34 at pH 7.4 (black bars), pH 6.5 (open bars) and reversible conditions (pH 6.5 to pH 7.4; grey bars), determined by analysis of 6 confocal microscopy fields. Error bars are derived from 3 independent experiments. **(C)** Expression of *CDKN1A*, *CCNB1* and *G0S2* was assessed by QRT-PCR in TG1 cells cultured for 5 days in proliferating (NS34 at pH7.4, black bars), quiescent (NS34 at pH 6.5, open bars) and reversible conditions (NS34 at pH 6.5 and then 5 days at pH 7.4, grey bars). Results are given relative to *TBP* (TATA-Box Binding Protein) expression level. Error bars are derived from 11 independent experiments (pH 7.4 and pH 6.5) and from 3 independent experiments (pH 6.5 to pH 7.4).

Figure S3. In vitro induction of quiescence in BTIC25 cells.

(A) BTIC25 cells were cultured for 5 days in NS34 medium at pH 7.5 (a) or pH 6.5 (b) to assess their ability to proliferate or to stop proliferation and go to quiescence. In NS34 medium, cells are floating and never form monolayer. **(B)** Cell proliferation measured by counting the number of viable cells over 5 days in NS34 at pH 7.4 in absence (black bars) or presence of SKF96365 (10 µM) (striped bars) and in NS34 at pH 6.5 (open bars) for TG1 cells (left panel) and for BITC25 cells (right panel). Each measure were done in duplicate and with 3 independent experiments. **(C)** Expression of *CDKN1A*, *CCNB1* and *G0S2* was assessed by QRT-PCR in TG1 cells after 5 days in NS34 at pH7.4 in absence (black bars) or presence of SKF96365 (10 µM) (striped bars) and in NS34 at pH 6.5 (open bars) for BITC25 cells. Results

are given relative to *TBP* (TATA-Box Binding Protein) expression level. Error bars are derived from 11 independent experiments. Error bars are derived from 3 independent experiments. Pictures were taken with a 20X 0.40 N.A. objective on Nikon eclipse TS100 microscope. Scale bars: 100 µm in A.

Figure S4. Remodeling of SOCE activity between proliferative and quiescent GSLCs.
(A) Structure of the chimeric gene encoding the CytGA probe. The open-reading frame (ORF) encoding the Green Fluorescent Protein (EGFP) is linked to the ORF encoding the apo-aequorin as described previously[1]. Confocal microscopy observation of the GFP signal of electroporated TG1 cells showed good expression of the probe in the cytosol (green). Nucleus was labelled with Draq5 (blue). Scale bar: 5 µm. **(B)** Structure of the chimeric gene encoding the MitGA probe. The open-reading frame (ORF) encoding the Green Fluorescent Protein (EGFP) is linked to the ORF encoding the apo-aequorin and to the mitochondrial targeting sequence Cox VIII. Confocal microscopy observation of the GFP signal of electroporated TG1 cells showed good expression of the probe in the mitochondria (merge). Nucleus was labeled with Draq5 (blue) and mitochondria with anti-TOM20 (red). **(C)** Representative PMT traces obtained from CytGA expressing TG1_C1 cells in proliferating (left panel, pH 7.4) or in quiescent (right panel, pH 6.5) conditions. Values are plotted as L/L_{TOTAL} and every trace is the mean of 3 independent experiments. Prior to recording, cells were washed with Ca^{2+}-free medium. The arrows indicate the time at which medium containing 1 mM Ca^{2+} is perfused. Pictures in A and B were taken with a 63X 1.40 N.A. objective on a Leica SP8 upright confocal microscope.

Figure S5. Calcium signaling is involved in the induced-quiescent state.
(A) Cell proliferation measured by counting the number of viable cells over 5 days in NS34 medium at pH 7.4 in absence (black bars) or presence of EGTA (5 or 10 µM) (grey bars). Each measure was done in triplicates; 3 independent experiments were performed. **(B)**. Expression of *CDKN1A*, *CCNB1* and *G0S2* was assessed by QRT-PCR after 5 days of culture in TG1 cells in NS34 at pH7.4 in absence (black bars) or presence of EGTA (grey bars) and compared to TG1 cells in NS34 at pH 6.5 (open bars). Results are given relative to *TBP* (TATA-Box Binding Protein) expression level. Error bars are derived from 11 independent experiments. **(C)** Expression of cyclins required during the different phases of the cell cycle; *CCND1*, *D3* (G1), *E2* (G1/S transition) and *A2* (S/G2 transition), of *PCNA* (Proliferating Cell Nuclear Antigen) and of *Hes1* which is known to be involved in the control of the reversibility of cellular quiescence. Expression of these cell cycle markers was assessed by QRT-PCR after 5 days of culture in TG1 cells in NS34 at pH7.4 in absence (black bars) or presence of EGTA (grey bars) and compared to TG1 cells in NS34 at pH 6.5 (open bars). Results are given relative to *TBP* (TATA-Box Binding Protein) expression level. Error bars are derived from 4 independent experiments. **(D)** Immunofluorescence for Ki67 of TG1 cells after 5 days in culture at pH 7.4 in presence of EGTA (10 µM). Nuclei were visualized by Draq5. Scale bar = 20 µm. **(E)** EdU incorporation (upper panels) and Ki67 positive cells (lower panels) at pH 7.4 and pH 6.5 and at pH7.4 in presence of SKF96365 (10 µM) for TG1 cells. Scale bar: 5 µm. Pictures taken with a 63X 1.40 N.A. objective on a Leica SP8 upright confocal microscope.

Figure S6. Expression of the members of the Store-Operated Calcium Entry.
Expression of *STIM* and *ORAI* family members in TG1 cells was assessed by QRT-PCR cultured for 5 days either in NS34 at pH7.4 (black bars, proliferative condition) or in NS34 at pH 6.5 (open bars, quiescent condition). Results are given relative to *TBP* (TATA-Box Binding Protein) expression level. Error bars are derived from 3 independent experiments.

Figure S7. Mitochondrial morphology remodeling in BTIC25 cells. Confocal microscopy analysis of mitochondria shapes in proliferating (left panel, pH 7.4), in quiescent BTIC25 cells (middle panel, pH 6.5) and in SKF96365-treated BTIC25 cells (right panel, pH 7.4 + SKF96365 10 µM). Mitochondria were labeled with TOM20 antibody (green) and nucleus with Draq5 (red). Tubular mitochondria are found in proliferating BTIC25 cells (left panel and Inset) and donut-shaped mitochondria in quiescent and SKF96365-treated BTIC25 cells (Middle and right panel). Pictures taken with a 63X 1.40 N.A. objective on a Leica SP8 upright confocal microscope. Scale bars: 10 µm.

Figure S1

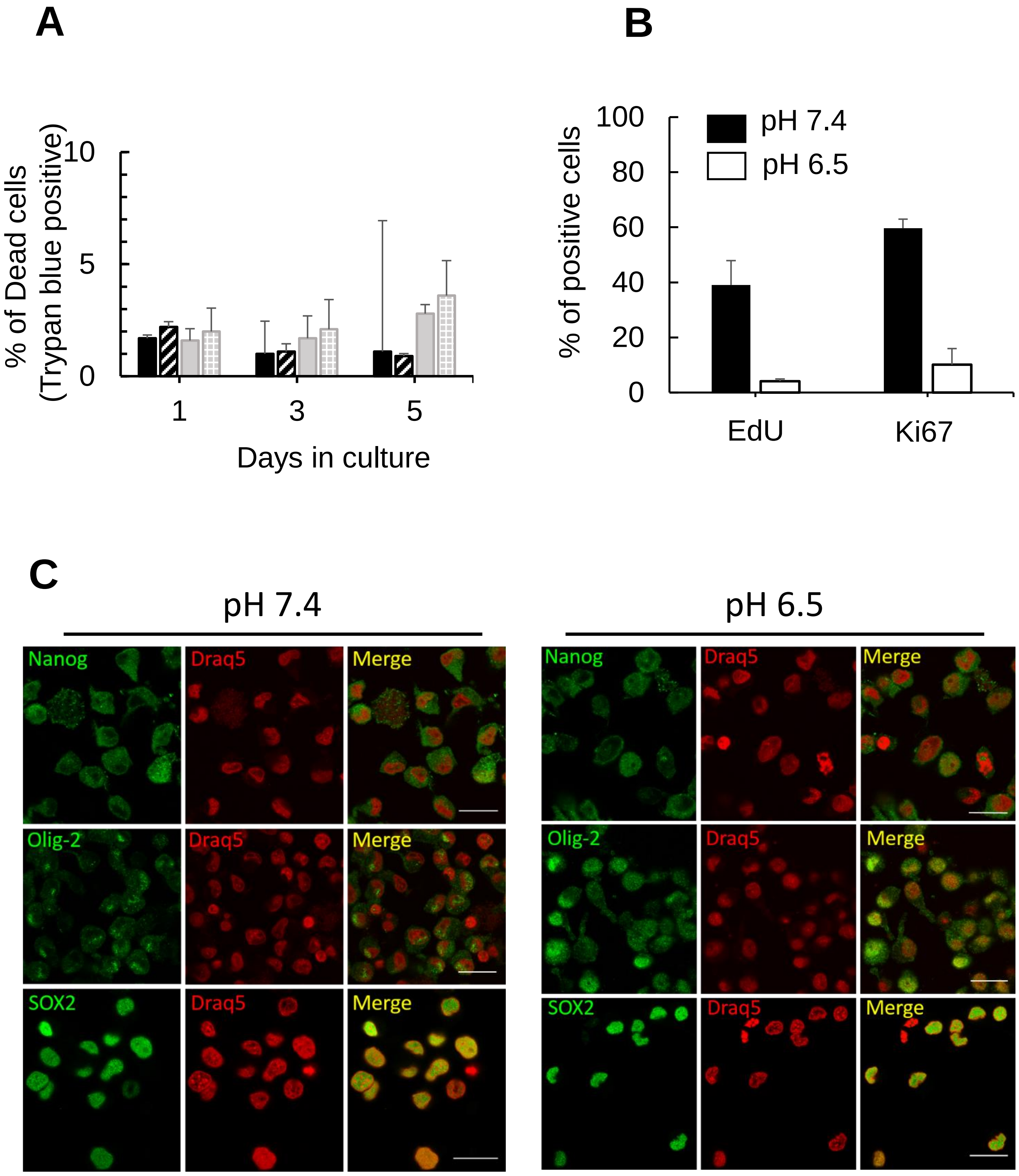
A
% of Dead cells
(Trypan blue positive)
10
5
0
1
3
5
Days in culture
B
% of positive cells
100
80
60
40
20
0
pH 7.4
pH 6.5
EdU
Ki67
C
pH 7.4
pH 6.5
Nanog
Draq5
Merge
Olig-2
Draq5
Merge
SOX2
Draq5
Merge
Nanog
Draq5
Merge
Olig-2
Draq5
Merge
SOX2
Draq5
Merge

Figure S2

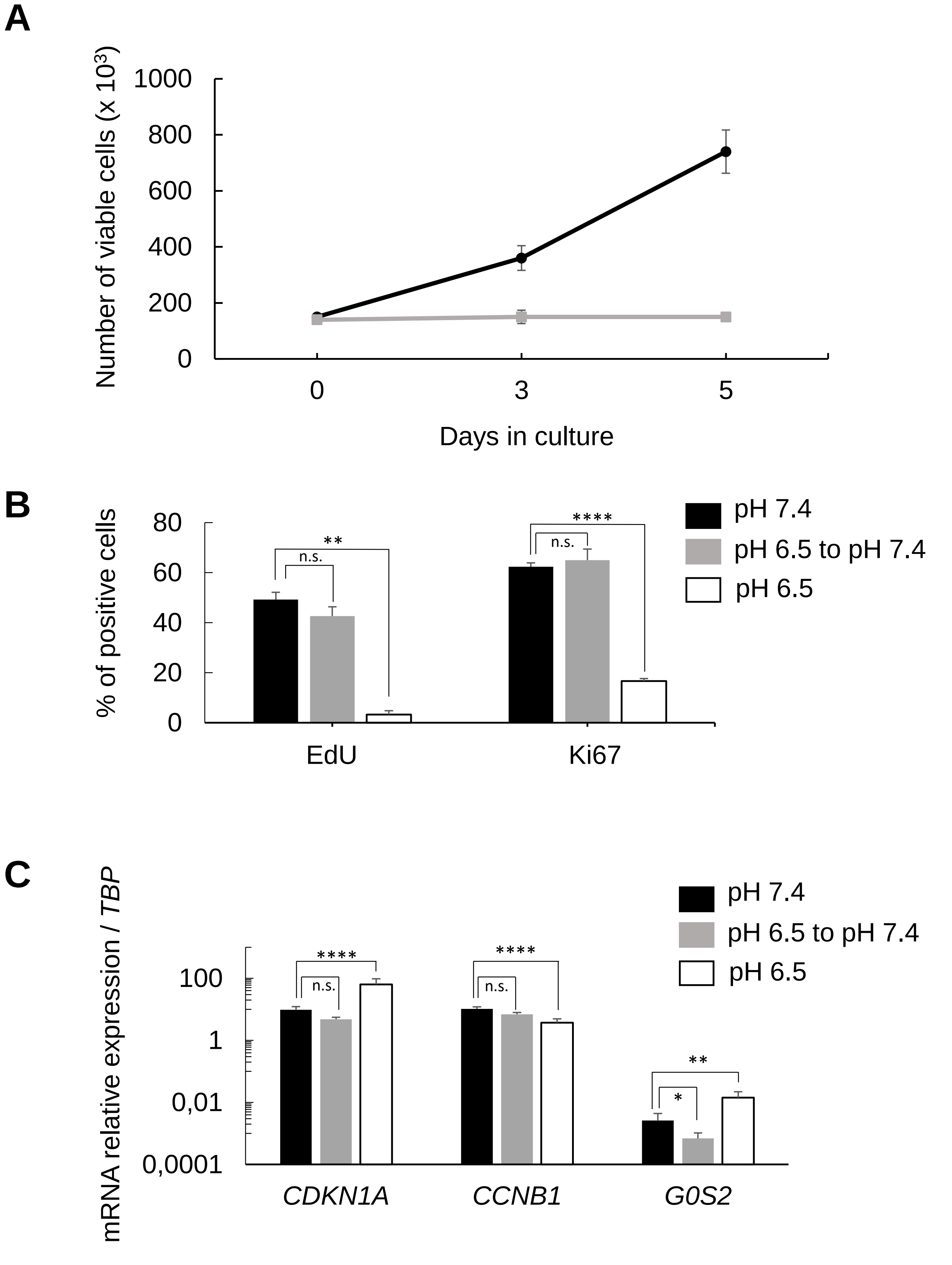
A
Number of viable cells (x 10^3)
1000
800
600
400
200
0
0
3
5
Days in culture
B
% of positive cells
80
60
40
20
0
**
n.s.
****
n.s.
EdU
Ki67
pH 7.4
pH 6.5 to pH 7.4
pH 6.5
C
mRNA relative expression / TBP
100
1
0,01
0,0001
****
n.s.
****
n.s.
**
*
CDKN1A
CCNB1
G0S2
pH 7.4
pH 6.5 to pH 7.4
pH 6.5

Figure S3

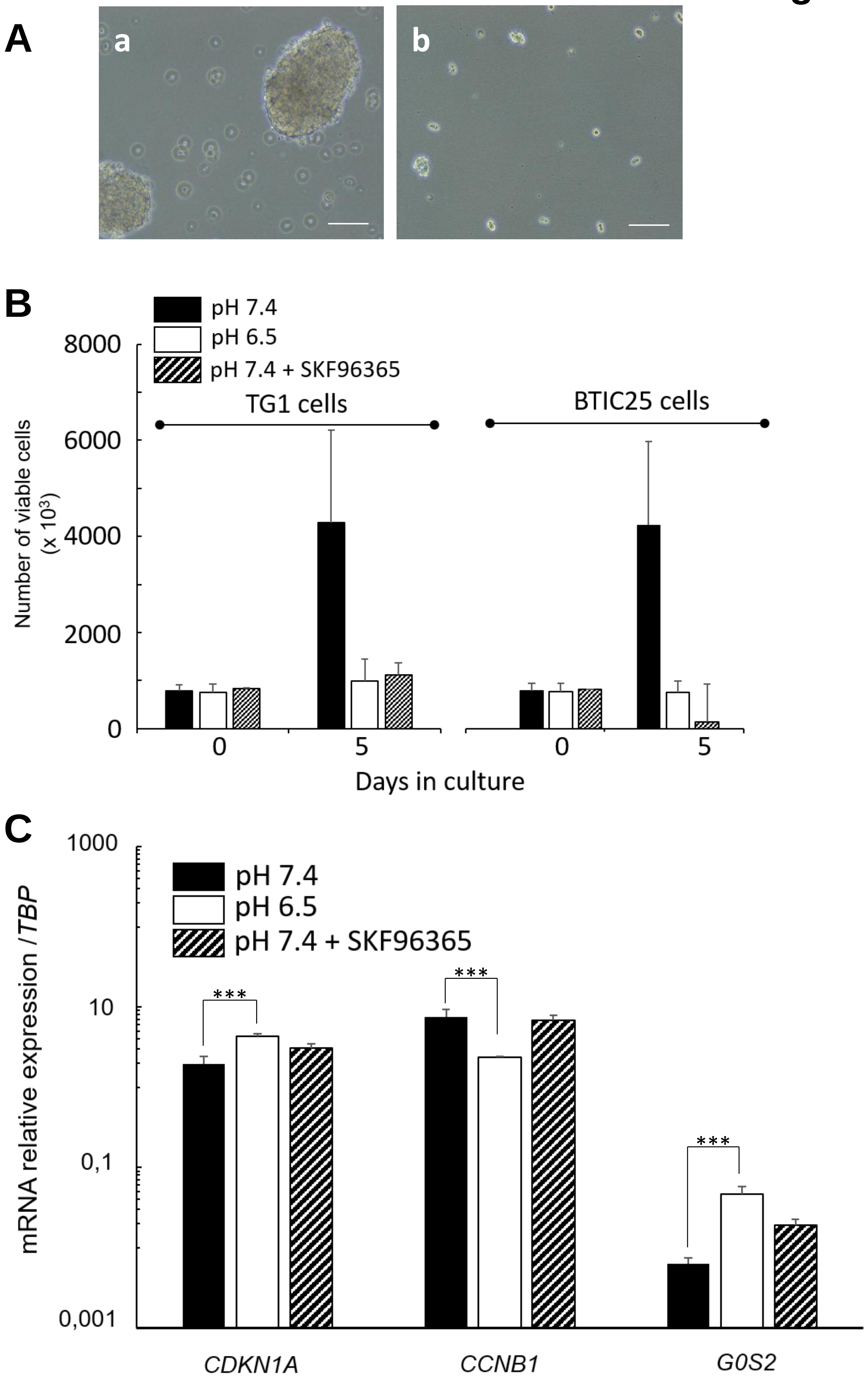
A
a
b
B
pH 7.4
pH 6.5
pH 7.4 + SKF96365
TG1 cells
BTIC25 cells
Number of viable cells
(x 10³)
8000
6000
4000
2000
0
0
5
0
5
Days in culture
C
mRNA relative expression /TBP
1000
10
0,1
0,001
pH 7.4
pH 6.5
pH 7.4 + SKF96365
***
***
***
CDKN1A
CCNB1
G0S2

Figure S4

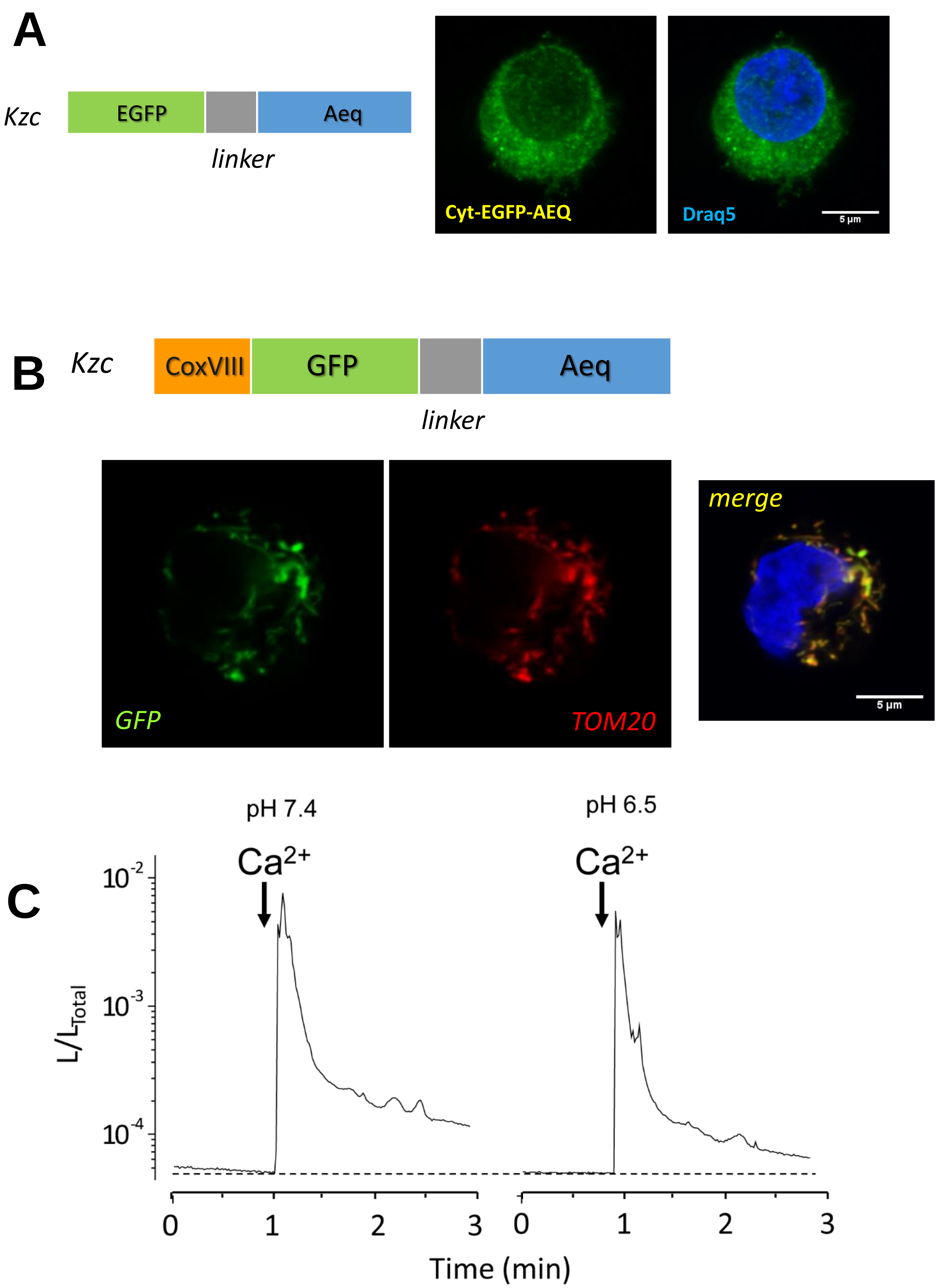
A
Kzc
EGFP
Aeq
linker
Cyt-EGFP-AEQ
Draq5
5 µm
B
Kzc
CoxVIII
GFP
Aeq
linker
GFP
TOM20
merge
5 µm
C
pH 7.4
pH 6.5
Ca2+
Ca2+
10-2
10-3
10-4
L/LTotal
0
1
2
3
0
1
2
3
Time (min)

Figure S5

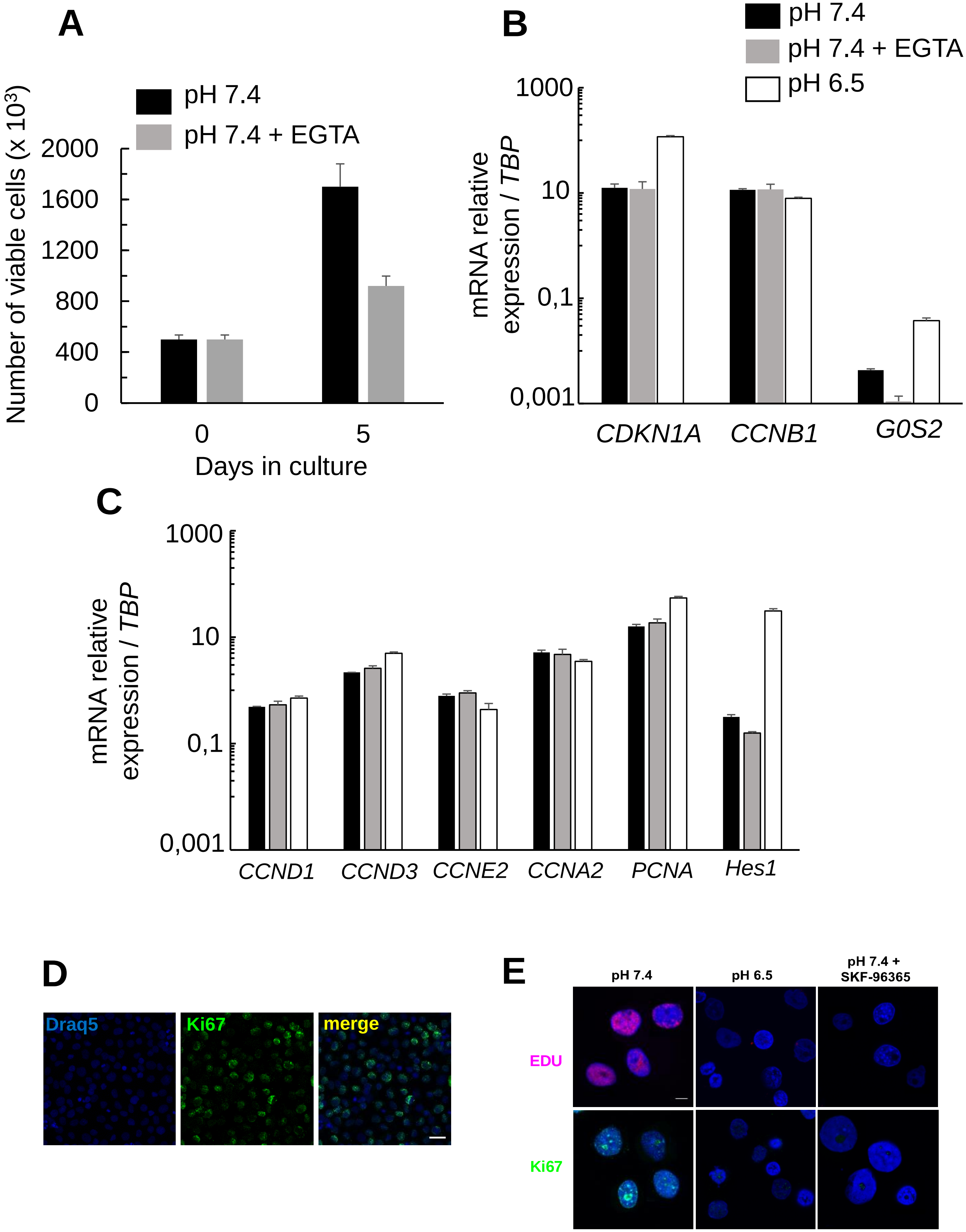
A
pH 7.4
pH 7.4 + EGTA
Number of viable cells (x 10³)
2000
1600
1200
800
400
0
0
5
Days in culture
B
pH 7.4
pH 7.4 + EGTA
pH 6.5
mRNA relative expression / TBP
1000
10
0,1
0,001
CDKN1A
CCNB1
G0S2
C
mRNA relative expression / TBP
1000
10
0,1
0,001
CCND1
CCND3
CCNE2
CCNA2
PCNA
Hes1
D
Draq5
Ki67
merge
E
pH 7.4
pH 6.5
pH 7.4 + SKF-96365
EDU
Ki67

Figure S6

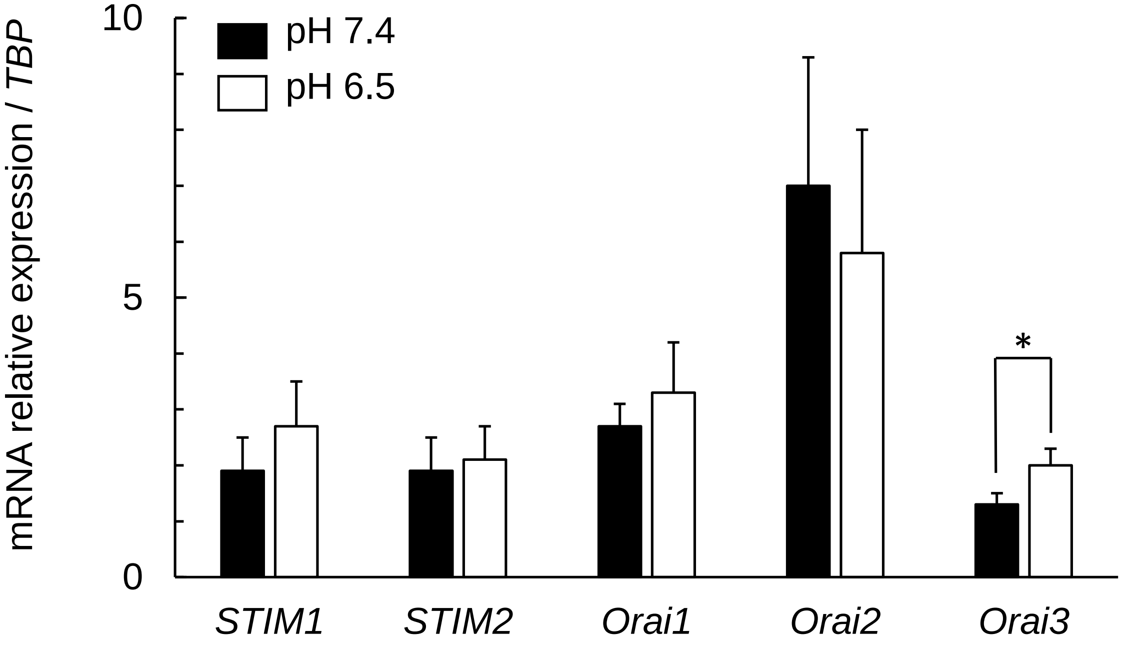

mRNA relative expression / TBP
10
5
0
pH 7.4
pH 6.5
*
STIM1
STIM2
Orai1
Orai2
Orai3

Figure S7

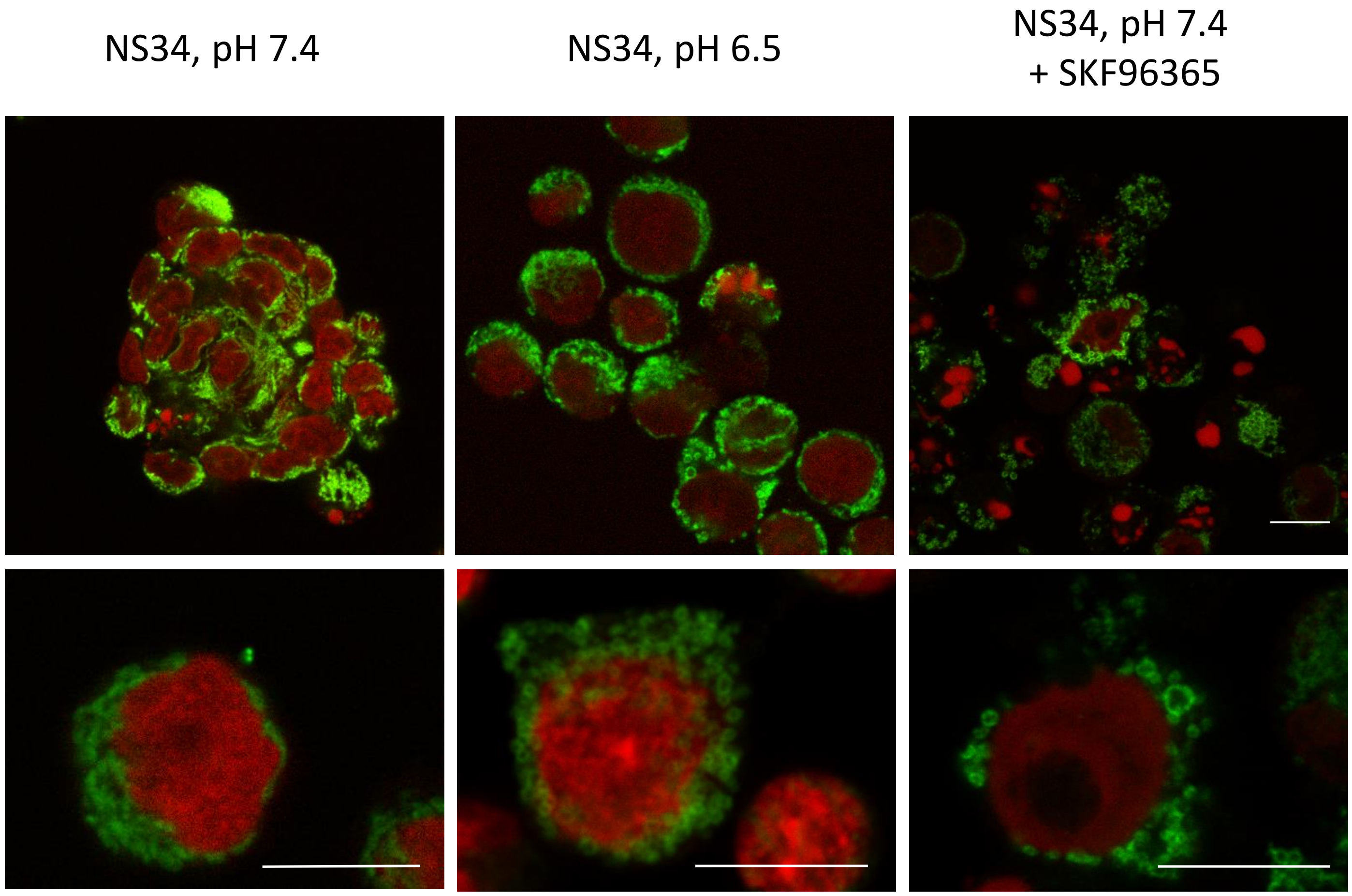

Supplement: Supplementary file 1 — supplementary information [file 41598_2018_28157_MOESM1_ESM.pdf]
